# The novel type II toxin–antitoxin PacTA modulates *Pseudomonas aeruginosa* iron homeostasis by obstructing the DNA-binding activity of Fur

**DOI:** 10.1093/nar/gkac867

**Published:** 2022-10-06

**Authors:** Yingjie Song, Siping Zhang, Zirui Ye, Yongyan Song, Lin Chen, Aiping Tong, Yongxing He, Rui Bao

**Affiliations:** Center of Infectious Diseases, State Key Laboratory of Biotherapy, West China Hospital, Sichuan University, Chengdu 610093, China; Central Laboratory, Clinical Medical College & Affiliated Hospital of Chengdu University, Chengdu 610081, China; Ministry of Education Key Laboratory of Cell Activities and Stress Adaptations, School of Life Sciences, Lanzhou University, Lanzhou 730000, China; Ministry of Education Key Laboratory of Cell Activities and Stress Adaptations, School of Life Sciences, Lanzhou University, Lanzhou 730000, China; Central Laboratory, Clinical Medical College & Affiliated Hospital of Chengdu University, Chengdu 610081, China; Central Laboratory, Clinical Medical College & Affiliated Hospital of Chengdu University, Chengdu 610081, China; Center of Infectious Diseases, State Key Laboratory of Biotherapy, West China Hospital, Sichuan University, Chengdu 610093, China; Ministry of Education Key Laboratory of Cell Activities and Stress Adaptations, School of Life Sciences, Lanzhou University, Lanzhou 730000, China; Center of Infectious Diseases, State Key Laboratory of Biotherapy, West China Hospital, Sichuan University, Chengdu 610093, China

## Abstract

Type II toxin–antitoxin (TA) systems are widely distributed in bacterial and archaeal genomes and are involved in diverse critical cellular functions such as defense against phages, biofilm formation, persistence, and virulence. GCN5-related N-acetyltransferase (GNAT) toxin, with an acetyltransferase activity-dependent mechanism of translation inhibition, represents a relatively new and expanding family of type II TA toxins. We here describe a group of GNAT-Xre TA modules widely distributed among *Pseudomonas* species. We investigated PacTA (one of its members encoded by PA3270/PA3269) from *Pseudomonas aeruginosa* and demonstrated that the PacT toxin positively regulates iron acquisition in *P. aeruginosa*. Notably, other than arresting translation through acetylating aminoacyl-tRNAs, PacT can directly bind to Fur, a key ferric uptake regulator, to attenuate its DNA-binding affinity and thus permit the expression of downstream iron-acquisition-related genes. We further showed that the expression of the *pacTA* locus is upregulated in response to iron starvation and the absence of PacT causes biofilm formation defect, thereby attenuating pathogenesis. Overall, these findings reveal a novel regulatory mechanism of GNAT toxin that controls iron-uptake-related genes and contributes to bacterial virulence.

## INTRODUCTION

Toxin–antitoxin (TA) systems are small genetic elements widely distributed among archaeal and bacterial genomes (1–3). TA systems generally encode a metabolically stable toxic protein and a cognate antitoxin. To date, TA systems are classified into type I–VIII, and of them, type II TA systems are the most abundant and extensively studied (1). In these systems, the toxins target essential cellular processes such as transcription or translation and cause growth arrest or cell death, while the antitoxins are noncoding RNAs or proteins that neutralize toxin activity (2). Most TA genes are genetically linked and organized within an operon, and their transcription are often regulated by ‘conditional cooperativity’ mechanism (1). Type I antitoxin genes encode small RNAs which could silence the transcripts encoding cognate toxins, while type V RNase antitoxins functions as endoribonuclease to specifically degrade transcripts of toxins (1). In type II TA systems, autoregulation of transcription is often mediated by the antitoxin that binds to TA operon promoter region while the toxin acts as a co-repressor (4,5). The type II TA complex is often a better repressor of transcription that the antitoxin alone (6,7). When the antitoxin is degraded, the transcription of toxin is increased and the released toxin exerts its effect on essential cellular process. Due to a substantial increase in the number of new type II TA members identified from different pathogens, the biological functions of the type II TA systems require to be further understood (4,5).

*P. aeruginosa* is among the main nosocomial pathogens that often causes chronic lung infections and readily acquires resistance to many antibiotics (8). It possesses various type II TA systems that potentially regulate stress tolerance and virulence (9,10). For instance, the HigBA system regulates persister formation under ciprofloxacin treatment (10), and the ParDE system confers resistance to quinolone and other antibiotics (11). Both these TA systems have been implicated in mediating biofilm formation and virulence. Nevertheless, the functions of most other type II TA systems in *P. aeruginosa* are unexplored. In this study, we focused on a hypothetical *P. aeruginosa* TA pair *pa3269*/*pa3270* that encodes a putative toxin PA3270 containing a GCN5-related N-acetyltransferase (GNAT) domain and a conserved acetyl-CoA-binding motif. This toxin has been hereafter referred to as PacT (*Pseudomonas* acetyltransferase toxin). GNAT is a large family of enzymes that transfers acetyl groups from acetyl-CoA to different substrates including antibiotics, polyamines, and proteins (12). Studies on GNAT-type toxins have revealed a common translation inhibition mechanism by acetylating amino-acylated tRNAs (aa-tRNAs), and all their cognate antitoxins possess a RHH (Ribbon-helix-helix) motif, and thus are termed as the GNAT-RHH TA system (13). However, PacT exhibited low homology with GNAT-RHH toxins and the cognate antitoxin PacA bears a HTH (helix-turn-helix)-XRE (xenobiotic response element) motif; thus, PacTA was classified as a GNAT-Xre TA system (14). These differences may contribute to the divergent functional roles of PacTA, but their biological significance needs to be fully defined.

Efficient iron uptake is critical for the survival, growth and virulence of *P. aeruginosa* since iron normally serves as a redox cofactor in many essential cellular processes, but excessive intracellular iron also cause hazardous effects (15). In order to control the intracellular iron content tightly, *P. aeruginosa* has evolved several distinct strategies to maintain iron homeostasis (13). One representative mechanism is the ferric uptake regulator Fur, a central transcriptional repressor that controls a large number of genes for iron acquisition and storage. Recent studies reveal that several type II TA systems are tightly associated with iron uptake process in bacteria, but there is still no evidence for their direct interactions (16,17). We here functionally validated the regulatory effects and physiological interaction of the PacTA system in *P. aeruginosa* PA14. Proteome analysis demonstrated that *pacTA* deletion led to downregulation of a series of iron-uptake-associated genes, resulting in reduced siderophore levels. A further study revealed that PacT could directly bind to the HTH domain of the key ferric uptake regulator Fur to attenuate its DNA-binding affinity. The expression of the PacTA module was induced under iron starvation, thus relieving the repression effect of Fur and increasing the synthesis of iron-uptake-related proteins. We also found that PacT homologs from other *Pseudomonas* species could bind to the Fur protein, suggesting that the direct PacT–Fur interaction is a common mechanism for PacT-type toxins. Moreover, the PacTA system contributed to biofilm formation and full virulence of *P. aeruginosa*, suggesting that this TA system is involved in host adaptation during infections. Taken together, the functional and structural characterizations of the PacTA GNAT-Xre TA pair provide new insights into the vastly diverse and complex type II TA systems.

## MATERIALS AND METHODS

### Bacterial strains and growth conditions

The *P. aeruginosa* strains derived from strain PA14 and plasmids used in this study are all detailed in Supplementary Table S1. The primers used in this study are detailed in Supplementary Table S2. β-galactosidase reporters were constructed by cloning gene promoters upstream of the *lacZ* in pRG970km, a derivative plasmid of pRG970. Complementation vectors were based on pME6032.

As for growth under different stress conditions, the *P. aeruginosa* PA14 strains were grown in Luria-Bertani (LB) medium at 37°C and then collected and treated with LB media containing 2 μg/ml gentamicin, 0.5 M NaCl, 1% v/v hydrogen peroxide, 1 mM 2,2′-dipyridine or transferred to M9 basic medium with 0.5% glucose, or left untreated for 2 hours.

### Sequence alignments and phylogenetic tree

The information of GNAT toxins and cognate antitoxins were obtained from the TADB 2.0 database (https://bioinfo-mml.sjtu.edu.cn/TADB2/index.php) (14). The amino acid sequence alignments were prepared using Clustal Omega (https://www.ebi.ac.uk/Tools/msa/clustalo) software. The phylogenetic tree of antitoxins from GNAT-Xre family was generated from PhyML 3.1/3.0 aLRT (http://www.phylogeny.fr/) (18) and refined graphically using Phylogenetic tree viewer (http://etetoolkit.org/treeview/) (19).

### β-Galactosidase assay

The putative promoter regions of *pacT* (P1) and *pacA* (P2) were predicted using Softberry online programs (20). The *higBA*, P1 and P2 promoters were cloned upstream of a promoterless *lacZ* gene in pRG970km to construct *lacZ* fusions (16). These vectors were first transformed into *Escherichia coli* S17-1 and mobilized into *P. aeruginosa* PA14 through incubating with S17-1. The colonies were screened in LB agar media containing kanamycin (120 μg/ml). The *P. aeruginosa* strains carrying the vectors were grown in LB medium as OD_600_ reached to 0.2, 0.4 and 0.8 at 37°C, respectively. Then the cells were collected and β-Galactosidase activities were measured as previously reported (16). β-Galactosidase activity was normalized by the β-galactosidase activity at the time of induction.

### Protein expression and purification

The *pacT* and *fur* genes were amplified from *P. aeruginosa* PA14 genomic DNA and subcloned into a pET-22b plasmid. *E. coli* BL21 (DE3) cells containing pET22b-*pacT* and pET22b-*fur*-TEV were grown at 37°C in LB media to an appropriate optical density and induced with 0.4 mM Isopropy-β-D-thiogalactoside (IPTG) for 16 hours at 16°C incubator shaker. The PacT proteins were purified in 25 mM Tris–HCl pH 7.5, 150 mM NaCl and concentrated to 14.8 mg/ml for following experiments. The Fur needs another high-salt elution (500 mM NaCl) to remove the nucleic acid contamination and TEV digestion to remove His-tag. As for the complex, the PacT_6×His_ and Fur were coincubated with Ni-agarose resins and then eluted with buffer containing 300 mM imidazole. The PacT homologous genes from *Pseudomonas jinjuensis* and *Pseudomonas flexibilis* were synthesized and subcloned into pET-22b, then expressed and purified similar to PacT to a final concentration at 7.8 mg/ml.

### Crystallization, diffraction data collection and structure solving

Crystallization was performed by sitting drop technique at 16°C. Crystals of apo-PacT were grown in 0.1% M *n*-octyl-β-d-glucoside, 0.1 M sodium citrate pH 5.5, and 20% PEG3350.Crystals of PT*_P. jinjuensis_* and PT*_P. flexibilis_* were grown in 0.1 M Tris–HCl pH8.5, 15% PEG6000 and 0.2 M NaCl, 0.1 M MES pH6.5, 10% PEG4000, respectively. X-ray data were collected at Shanghai Synchrotron Radiation Facility (SSRF) beamline BL-19U, then processed and solved via the molecular replacement method with previous structure (PDB: 1YRE) as template. The final structure data were listed in Supplementary Table S3.

### Construction of *P. aeruginosa* mutants

As previously reported, we used the two-step allelic exchange method to construct *P. aeruginosa* mutant strains (21). The upstream (800 bp) and downstream (800 bp) fragments of *pacT*, *pacA* or *pacTA* from the *P. aeruginosa* PA14 were subcloned into the suicide plasmid pEX18Gm. These vectors were first transformed into *E. coli* S17-1 and mobilized into *P. aeruginosa* PA14 through incubating with S17-1. The colonies were screened using sucrose-mediated counter-selection and further identified by PCR.

### Total protein extraction and bicinchoninic acid (BCA) protein assay

Protein was collected, reduced and alkylated from wild-type, Δ*pacA* and Δ*pacTA* strains according to a protocol of Wang *et al.* (22). The total purified peptides were solubilized in 20 μl of 0.1% formic acid (FA, v/v) and the concentration was measured using the BCA method, with minor modifications (23). Briefly, a standard curve was prepared by diluting BCA stock solution stock (5 mg/ml) with varying amounts of 0.1% FA, according to the manufacturer's instructions (Thermo Fisher Scientific). 20 μl of each sample was then mixed with 200 μl working reagent and incubated at room temperature for 30 min. The absorbance of the solutions at 570 nm was then measured using a full-wavelength microplate reader (Bio-Rad Laboratories, CA, USA). The processing flow of *E. coli* BL21 (DE3) strains carrying *pacT* and empty vector were consistent with *P. aeruginosa*.

### Mass spectrometry analysis

Approximately 2 μg samples were analyzed by orbitrap Fusion Lumos MS (Thermo Fisher Scientific, Waltham, MA, USA) coupled online to an EASY-nLC 1200 system with a data-independent acquisition mode (DIA) (24). The mobile phase used for the liquid chromatography consisted of buffer A (0.1% FA) and buffer B (80% acetonitrile, 0.1% FA). The peptides were separated using a 160-min non-linear gradient consisting of 4–35% buffer B for 85 min, 35–100% buffer B for 60 min and 100% buffer B for 15 min at a flow rate of 300 nl min^−1^. The acquired DIA data were analyzed by DIA-NN software against the protein database of *P. aeruginosa* PA14 (25) as well as *E. coli* BL21 (DE3). Cysteine carbamidomethylation was searched as the fixed modification, methionine oxidation was collected as the variable modification. Data processing was conducted using the software Perseus. Student's *t*-test was used to assess the significance of differential expression of proteins (DEPs) between two groups. Proteins that have significance level of *P* < 0.05 and fold change >1.5 or <–1.5 were considered as DEPs (detailed data in Supplementary Tables S4–S7).

### Electrophoretic mobility shift assays

Fur and PacT were incubated with DNA fragments generated by PCR (Supporting Supplementary Table S2) in 20 μl of gel shift-loading buffer (25 mM Tris–HCl pH 7.5, 150 mM NaCl) (16). Following incubation at 4°C for 30 min, the samples were analyzed by 10% polyacrylamide gel electrophoresis in 0.5× TBE (Tris/boric acid/EDTA) buffer at 120 V for 90 min. The bands were visualized under BioRad imager with Alex488 suite.

### RNA preparation and qRT-PCR analysis

The bacterial RNA used in this work was extracted using Trizol (Invitrogen, USA) (26). PrimeScript™ RT reagent Kit (TaKaRa, Beijing) was used to synthesize the cDNA and 2× ChamQ™SYBR^®^qPCR Master Mix (Vazyme, Nanjing) was used to perform qPCR assays. The detailed reaction system was 2× ChamQ SYBR qPCR Master Mix (Without ROX) 10 μl, Primer 1 (10 μM) 0.4 μl, Primer 2 (10 μM) 0.4 μl, Template cDNA 1 μg and ddH_2_O up to 20 μl. The detailed qPCR procedure used in study was: stage 1 (95°C 30 s, repeat once), stage 2 (95°C 10 s, 60°C 30 s, repeat for 40 times) and stage 3 (95°C 15 s, 60°C 60 s, 95°C 15 s, repeat once). The *oprL* gene was used as a normalizer. As for RT-PCR, the *pacTA* transcripts were amplified using cDNA synthesized from total RNA of *P. aeruginosa* as template, and the PCR products were sent to the company (TsingkeBiotechnology Co., Ltd.) for sequencing. All the primers used in this study were detailed in Supporting Supplementary Table S2.

### Siderophore purification

Briefly, *P. aeruginosa* strain PA14 and mutants were grown in LB media at 37°C overnight, then diluted in fresh LB at a ratio of 1:100 and grown at 37°C for 12 h (16). The cells were collected and removed by centrifugation to measure the supernatant at 400 and 600 nm absorbance. The final siderophore value was *A*_400_/OD_600_.

### Measurement of pyocyanin production

Pyocyanin was extracted from culture supernatants of *P. aeruginosa* and measured as previously reported (27). Briefly, 5 ml *P. aeruginosa* was grown at 37°C overnight and the supernatant was collected through centrifugation. Then 2 ml chloroform was added to 4 ml supernatant for extraction. The chloroform layer was transferred to a fresh tube and mixed with 0.66 ml 0.2 N HCI. After centrifugation, the top layer was selected for *A*_520_ measurement. Pyocyanin concentration was calculated as (*A*_520_/OD_600_) × 17.042.

### Immunoblotting

Proteins samples separated by 15% SDS-PAGE and blotted to a PVDF membrane. Membranes were blocked for 1 h with 5% not-fat milk in TBST buffer, and probed 1 hour at 4°C either in TBST with a 1:2000 anti-Flag antibody (ZEN-BIOSCIENCE, Catalogue No. 700002), or with a 1:1000 anti-His tag antibody (ZEN-BIOSCIENCE, Catalogue No. 251784). After washing by TBST buffer three times, membranes were incubated for 1 h with HRP-conjugated secondary antibodies (1:10000, Goat Anti-Rabbit IgG H&L (HRP), ZEN-BIOSCIENCE, Catalogue No. 511203) in TBST buffer. Chemiluminescence substrate (Abbkine, Catalogue No. K22020) was added and signal quantifications were done in BioRad imager. As for the acetylation assay, the PVDF membranes were firstly probed by Acetylated Lysine Antibody (Affinity, Catalogue No. DF7729), then incubated with Goat Anti-Rabbit IgG (H + L) HRP (Affinity, Catalogue No S0001).

### Biofilm formation assay

Biofilm was determined according to a previously described method (16). Briefly, overnight bacterial cultures were diluted at a ratio of 1:100 in LB medium and transferred into a 24-well PVC plate (Sigma) and incubated at 37°C for 48 h. Then the biofilm was stained with crystal violet and measured at 570 nm. Results are reported from three independent experiments with three replicates per experiment.

### *G. mellonella* killing assays

In brief, the *P. aeruginosa* PA14 WT and mutant strains were grown in LB media to an optical density at 600 nm (OD_600_) of 0.5–0.6, then collected and washed 3 times in sterile PBS (28). Each *G. mellonella* was injected with 10 μl *P. aeruginosa* dilution (5 × 10^3^ CFU/ml, serial dilution and plate counts) by 50-μl Hamilton syringe. Control group of *G. mellonella* was injected with 10 μl sterile PBS. Then the injected *G. mellonella* were incubated at 37°C and monitored in the next 30 h in 37°C incubator.

## RESULTS

### *P. aeruginosa* PacTA locus represents a novel GNAT toxin–antitoxin module

**Figure 1.**
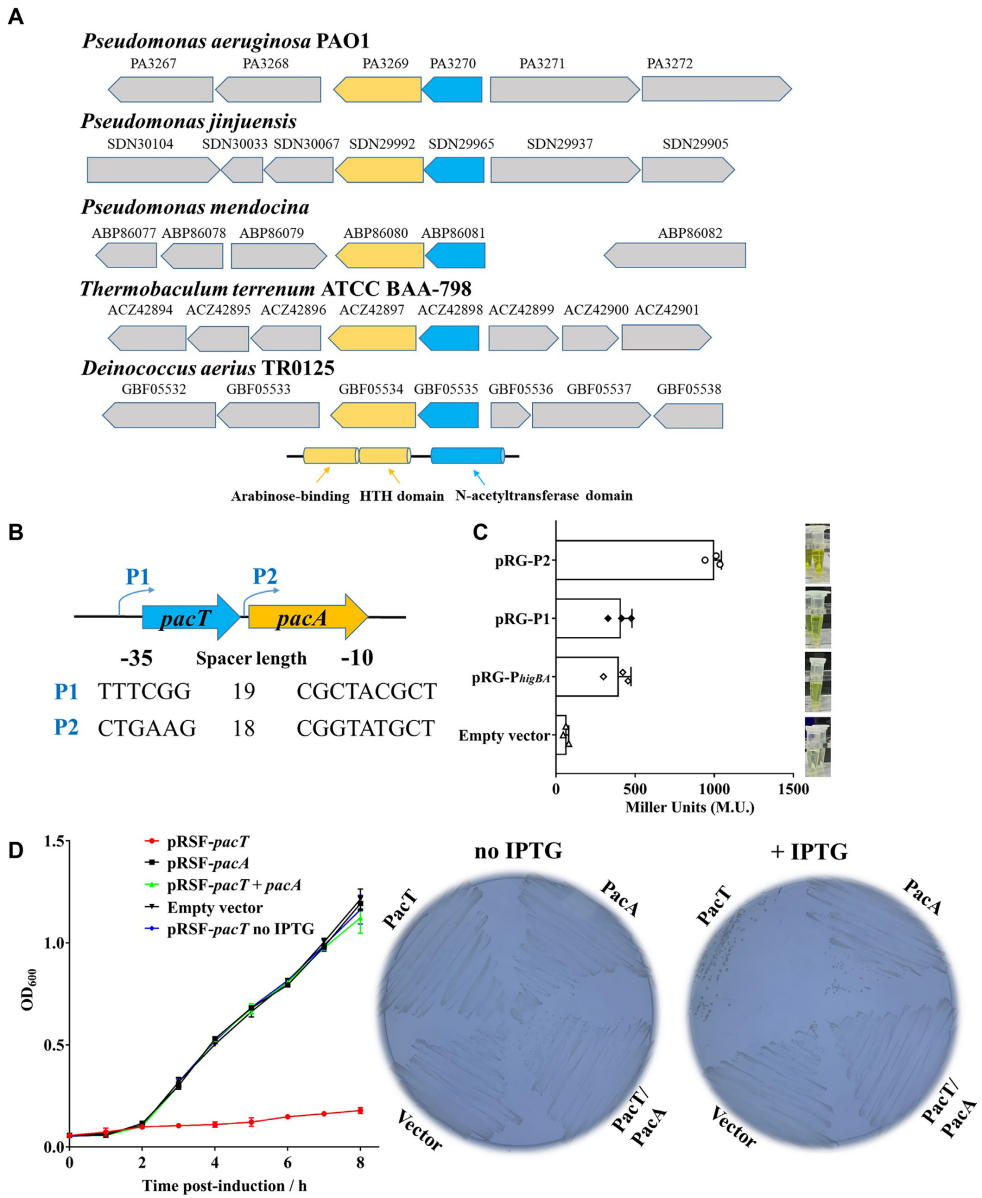
PacT/PacA is a conserved toxin-antitoxin module of GNAT/AraC family. (**A**) Schematic representation of the operon and surrounding genomic loci of the TA systems belong to the PacT/PacA family in different bacteria. (**B**) There are two separate promoters in *pa3270*/*pa3269* loci. The putative –35 and –10 regions of the two promoters are predicted by using Softberry online programs (http://linux1.softberry.com/) and underlined. (**C**) Construction of β-galactosidase reporter system to determine the promoter activities. The *higBA*, P1 and P2 promoters were cloned ahead of a promoterless *lacZ* gene in pRG970km to construct *lacZ* fusions and then transformed into *P. aeruginosa* PA14. The *P. aeruginosa* strains carrying the vectors were grown in LB medium as OD_600_ reached to 0.8 at 37°C. Then the cells were collected and β-Galactosidase activities were described in methods. (**D**) Expression of the toxin causes distinct growth arrest of *E. coli*, and the antitoxin PacA can neutralize the toxic effects. Overnight cultures of *E. coli* BL21 (DE3) carrying *pacT*, *pacA* or *pacTA* were reinoculated into fresh LB at OD_600_ = 0.05 containing 0.1 mM IPTG, and continued to cultivate at 37°C for growth detection. The experiments were repeated three times.

GNAT–RHH toxins, including *E. coli* AtaT and ItaT and *S. Typhimurium* TacT, have previously been reported to inhibit translation by acetylating charged tRNAs (13,29,30). These experimentally verified GNAT–RHH toxins only share 23.1%–25.53% identities with PacT, although all of them exhibit a conserved GNAT-fold and contain a unique motif (T/L-X-L/V/I-X_5_-G/K-X-G-L/F/W) for acetyl-coenzyme A binding (Supplementary Figure S1). The sub-classification of the GNAT TA systems was based on the antitoxin domain characteristics. One unique feature of the GNAT-Xre antitoxin is the additional domain apart from the Xre-HTH DNA-binding module. The overall phylogenetic structure of GNAT-Xre antitoxins from the TADB 2.0 database revealed that PacA represents a unique subgroup different from the remaining members (14), containing an N-terminal arabinose-binding domain and a C-terminal HTH domain (Figure 1A and Supplementary Figure S2).

Similar with the *P. aeruginosa* HigBA TA system, all PacTA-like TA systems are adapted to the ‘reverse’ genetic arrangement in which the antitoxin is located downstream of the toxin, and two putative promoters are present in the upstream of *pacT* and the intergenic region between *pacT* and *pacA* (Figure 1B) (16). To evaluate the promoter activity, we generated β-galactosidase reporter gene constructs by fusing these two promoter regions with a promoterless *lacZ* gene and performed the *lacZ* reporter assay in *P. aeruginosa* (16). As shown in Figure 1C and Supplementary Figure S3A, both the P1 and P2 promoters showed β-galactosidase activities comparable to that of the P*_higBA_* promoter, and P2 exhibited higher activity than P1. Further RT-PCR assay demonstrated that *pacT* and *pacA* could be co-transcribed in *P. aeruginosa*, this is consistent with the *mqsRA* and *higBA* operons, in which the additional promoters allow excess antitoxin production (Supplementary Figure S3B) (7,31). We subsequently investigated the possible toxic effect of PacT by inducing its expression in *E. coli* and *P. aeruginosa*. A remarkable growth arrest of cells was observed, which could be rescued by simultaneously overexpressing PacA (Figure 1D and Supplementary Figure S3C and D). By contrast, the antitoxin PacA and empty vector had no toxic effects.

**Figure 2.**
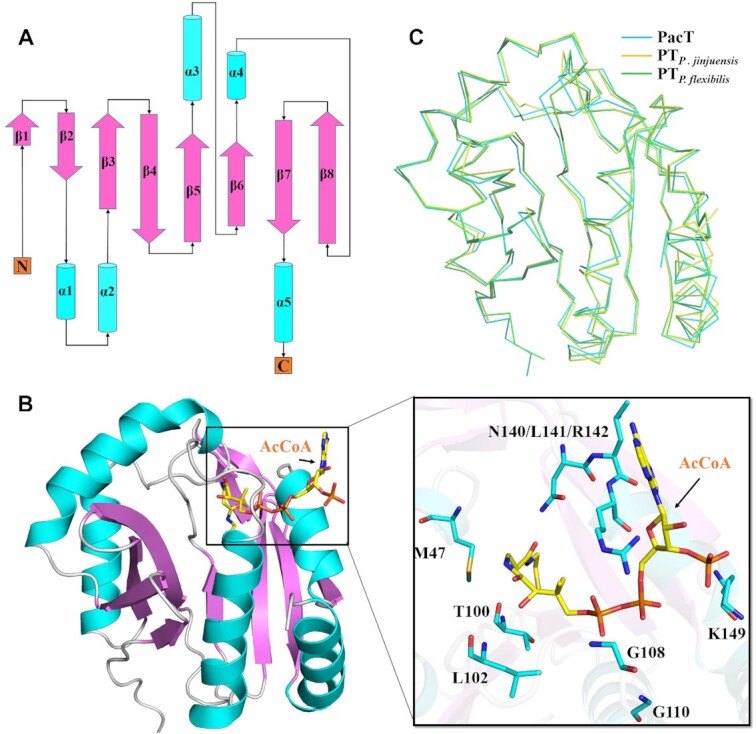
Overall structures of PacT and homologues from other Pseudomonas species. (**A**) Topological diagram of the PacT structure. (**B**) Orthogonal view of PacT (cartoon) bound to AcCoA (sticks), the AcCoA is aligned to the apo structure of PacT through superposition with the previous reported structure (PDB: 1YRE). The right panel shows the detailed interaction between PacT and AcCoA, and the resides are shown in sticks. (**C**) Structural comparisons between PacT (cyan), PT*_P. jinjuensis_* (yellow) and PT*_P. flexibilis_* (green).

To elucidate the structural mechanism of PacT, we have also solved crystal structures of PacT and homologues from *P. jinjuensis* and *P. flexibilis* through molecular replacement with PacT from *P. aeruginosa* PAO1 as template (PDB: 1YRE) (Supplementary Table S3). The apo-form PacT exhibits a typical GNAT family fold consisting with a central, eight mixed β sheets which besieged by two or three α helices on each side (Figure 2A and B). Overall structure of PacT in this work is highly similar with the previously reported structure of PacT bound with AcCoA ligand (PDB: 1YRE), and the structure superposition revealed that the resides including M47, T100, L102, G108, G110, N140, L141, R142 and K149 are involved in the interaction with AcCoA (Figure 2B). In addition, both the structures of PacT homologues from *P. jinjuensis* and *P. flexibilis* are in complex with AcCoA, and share similar fold with PacT in superposition (mean RMSD 0.28Å, Figure 2C). Taken together, these results confirmed that the PacT belongs to GNAT family and the PacTA constitutes a typical type II TA pair.

### PacT overexpression inhibits translation in *E. coli*

**Figure 3.**
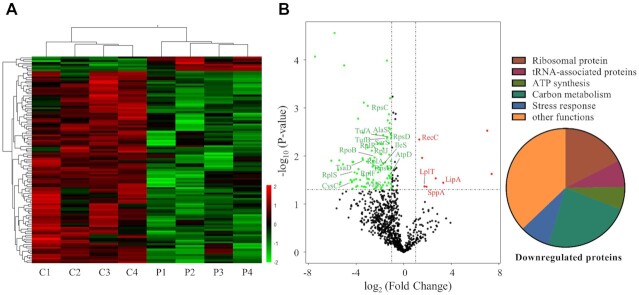
Translation inhibition by overexpressing PacT toxin. (**A**) Hierarchical clustering of the z-scored extracted ion chromatogram was used to evaluate the reproducibility of the proteome quantification in PacT overexpression strain. Overnight cultures of *E. coli* BL21 (DE3) carrying *pacT* or empty vector was reinoculated into fresh LB at OD_600_ = 0.05 and continued to cultivate at 37°C when OD_600_ reached to 0.6. Then the PacT was induced by adding 0.4 mM IPTG for another 6 h at 37°C. All the cells were collected for MS analysis. C: control, *E. coli* harboring empty vector. P: *E. coli* harboring vector for PacT expression. (**B**) Volcano plot displaying the proteomic profiles of control and PacT overexpression strains. The significantly up and down regulated proteins are labeled with red and green, respectively. The downregulated proteins were also categorized by functional category.

To investigate how PacT exerts its toxic effect in bacteria, we performed global proteomic profiling of a PacT overexpression strain and compared it with the parental *E. coli* strain. Of the 881 proteins identified, 105 proteins were differentially expressed between the WT and PacT-overexpressing strains, with most of them exhibiting reduced expression levels (Figure 3A and B, Supplementary Table S4). A majority of the identified proteins with reduced expression levels participated in known functions and were mapped to carbon metabolism and protein synthesis, especially synthesis of ribosomal protein subunits such as 50S ribosomal protein RplS, 50S ribosomal protein RplF, 30S ribosomal protein S3, 50S ribosomal protein RpsC, 30S ribosomal protein RpsO, and 30S ribosomal protein RpsJ. This is in agreement with the downregulation of ribosomal content and protein translation in the presence of GNAT toxins (29). Moreover, a significant decrease was observed in the expression of proteins involved in aa-tRNA biosynthesis, including serine-tRNA ligase SerS, isoleucine-tRNA ligase IleS, aspartate-tRNA ligase AspS, and valine-tRNA ligase ValS. Additionally, the expression of the elongation factors TufA and TufB, which directly promote the binding of aminoacyl-tRNA to the A-site of ribosomes during protein biosynthesis, was also decreased (32). The expression of a relatively smaller group of proteins, such as lipoyl synthase LipA, DNA repair enzyme RecC, lysophospholipid transporter LplT, and peptide peptidase SppA, was significantly increased in the PacT-overexpressing strain. These upregulated proteins participate in various cellular processes and might be partly associated with the downstream regulatory effects of PacT. Taken together, these observations suggest that PacT can repress the global translation process in bacteria.

### Proteomic analysis of PacTA deletion mutants reveals its roles in bacterial iron metabolism

**Figure 4.**
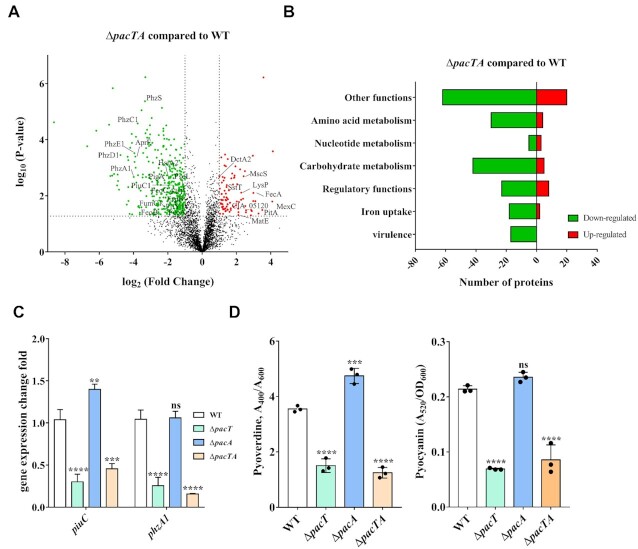
Downregulated iron uptake associated proteins caused by deletion of *pacTA*. (**A**) Volcano plot displaying the proteomic profiles of WT and *ΔpacTA* strains. Overnight cultures of WT and *ΔpacTA* strains were reinoculated into fresh LB at OD_600_ = 0.05 and continued to cultivate at 37°C when OD_600_ reached to 0.6–0.8, then the cells were collected for MS analysis. The significantly up and down regulated proteins are labeled red and green, respectively. (**B**) The number of increased and repressed proteins in (A) was categorized by functional category. (**C**) The expression levels of *piuC* and *phzA1* of Δ*pacT*, Δ*pacA* and Δ*pacTA* strains were measured by qRT-PCR, respectively. The bacterial culture method was the same as that in Figure 3A. The *oprL* gene was used as a normalizer. (**D**) Pyoverdin and pyocyanin production of Δ*pacT*, Δ*pacA* and Δ*pacTA* strains. *P < 0.05; **P< 0.01; ***P< 0.001 and ****P < 0.0001 by one-way ANOVA statistical test.

To characterize the role of the PacTA pair in *P. aeruginosa*, we performed global proteomic profiling of the Δ*pacA*, Δ*pacTA*, and WT strains by using label-free quantitative proteomics. Compared to WT, the whole proteins of *P. aeruginosa* Δ*pacA* presented a decreasing trend. Of the 3576 proteins, 46 proteins were significantly down-regulated and 4 proteins were up-regulated (Supplementary Figure S4A and 4C, Supplementary Table S5). Among the 46 down-regulated proteins, a number of proteins are involved in carbon metabolism, nucleotide metabolism and amino acid metabolism, including serine 3-dehydrogenase (CIA_01171), 2-methylcitrate dehydratase PrpD, succinate dehydrogenase SdhD, 3-hydroxybutyrate dehydrogenase BdhA, amino acid dehydrogenase CIA_03490, 2,3-butanediol catabolism dehydrogenase CIA_04250, Glycolate oxidase subunit GlcF, formiminoglutamate deiminase HutF, aspartate aminotransferase IlvE, nucleotide hydrolases dgt2, 3-butanediol catabolism dehydrogenase CIA_04250. Furthermore, the proteins involved in transport process were also reduced, including peptide transporter CIA_00319, ion channel protein MscS, potassium transporter KdpB, MFS family protein CIA_03970. This further proves that PacT could significantly affect the bacterial growth and protein expression. As for *P. aeruginosa* Δ*pacTA*, 3575 proteins were identified, in which 91 proteins were upregulated and 385 were downregulated compared with the WT strain (Figure 4A and Supplementary Figure S4B, Supplementary Tables S6 and S7). Furthermore, functional enrichment analysis was performed for these proteins (Figure 4B). Among the repressed proteins, a majority of the identified proteins were involved in carbohydrate and amino acid metabolisms. Moreover, a larger number of proteins with reduced expression levels were predicted to play vital roles in virulence. These included components of the type VI secretion system (T6SS) (HsiA2, HsiB2, HsiC2, ClpV1, HsiJ2, Hcp2) (33), alkaline protease secretion protein (AprA–AprL) (34), and flagellar proteins PilY1 (35) and FliT (36). Of the proteins with elevated expression levels, approximately 15% of the proteins belonged to the membrane transporter family (Figure 4A), including lysine transporter LysP, multidrug efflux MexC, ABC transporter permease MlaE, ion channel protein MscS, C4-dicarboxylate ABC transporter DctA_2_, and multidrug transporter MatE. In addition, numerous iron-uptake-associated proteins exhibited significantly reduced expression levels (Figure 4A). For instance, PKHD-type hydroxylase PiuC (15), fumarate hydratase FumC1 (15), ferrous iron transport protein FeoA (37), ferrous iron transport protein FeoB, ferrous iron transport protein FeoC, and heme oxygenase PigA (38) are key factors promoting bacterial iron absorption. Furthermore, evidence has shown that CIA_04582 (PA0473) and CIA_05976 (CbpA) also contribute to iron homeostasis in *P. aeruginosa* (39,40). The expression of PhzA-G involved in the biosynthesis of pyocyanin, a pigment with antibiotic activity, was significantly reduced in the Δ*pacTA* strain (41). These observations suggest that the PacTA system plays a vital role in iron uptake and pyocyanin biosynthesis.

To validate the aforementioned assumption, *piuC* and *phzA1* were selected to test and verify their expression patterns in Δ*pacT*, Δ*pacA* and Δ*pacTA*. The expression levels of both *piuC* and *phzF1* were notably reduced in the absence of *pacT* rather than *pacA* (Figure 4C). In concordance, siderophore and pyocyanin production was remarkably decreased in Δ*pacT* and Δ*pacTA* strains compared with the WT strain, while no significant changes were observed in the Δ*pacA* strain (Figure 4D, Supplementary Figure S5A and B). Taken together, these results verified the vital roles of the PacTA system in iron uptake.

### PacT binds to the global regulator Fur and inhibits its DNA-binding activity

**Figure 5.**
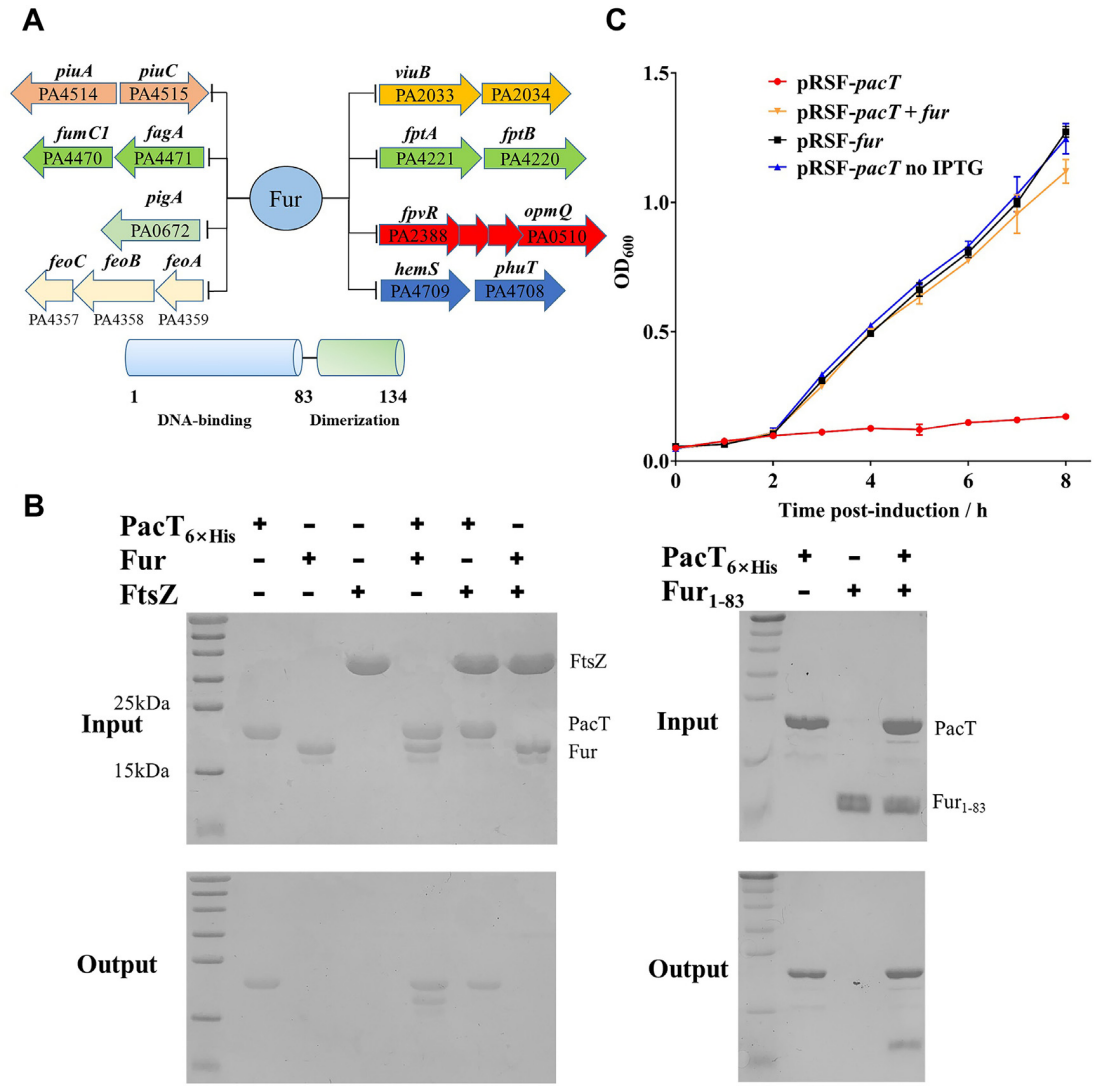
PacT binds to the DNA-binding domain of Fur. (**A**) Genes involved in iron uptake, transport and storage are under control of Fur. Fur consists of a DNA-binding domain (residue 1 to residue 83) and a C-terminal domain (residue 84 to residue 134). (**B**) The interaction between Fur and PacT was performed by pull-down assays. 20 μl His6-PacT (PacT_6×His_, 20 μM) was incubated with 10 μl full-length Fur, Fur_1–83_ or FtsZ (35 μM) without His tag. The protein complexes were captured by Ni-agarose resins and then detected by SDS-PAGE. The FtsZ served as negative control. (**C**) Co-expression of Fur could neutralize the toxic effects caused by PacT. The overnight cultures of *E. coli* strains harboring *pacT*, *fur* and *pacT*-*fur* were reinoculated into fresh LB with 0.1 mM IPTG at OD_600_ = 0.1 and grown at 37°C in the LB medium, the growth curves were detected to analysis the neutralization upon PacT. The experiments were repeated three times.

Regulation of iron uptake by type II TA systems such as *P. aeruginosa* HigBA and *Deinococcus radiodurans* MazEF have been investigated previously (16,42), however, the regulatory functions in iron uptake have not been reported for GNAT toxins. In *P. aeruginosa*, the major intracellular iron response regulator is Fur, which is also a conserved global regulator across many bacteria (15). Fur consists of a N-terminal DNA-binding domain that represses target gene expression by directly binding to the operators and a C-terminal domain that mediates dimerization by employing iron cofactors (Figure 5A) (43). Fur is well-known to modulate essential iron uptake processes including siderophore biosynthesis and iron transport (44). For example, ferric-chelate reductase ViuB, outer membrane protein OpmQ, Fe^3+^-pyochelin receptor FptA, and hemin-degrading factor HemS are key factors promoting bacterial iron absorption (Figure 5A); Fur directly represses operons encoding all these proteins (15). In our study, several iron-uptake-related proteins downregulated in the Δ*pacTA* strain, including *piuC*, *fumC1*, *pigA and feoA/B/C*, are also direct downstream targets of Fur (15). This prompted us to further investigate the relationship between PacT and Fur.

Recombinant PacT (tagged 6 × His-tag) and Fur (His-tag removed by TEV protease) were purified and subjected to *in vitro* binding assays. According to the results, PacT directly bound to Fur (Figure 5B, Supplementary Figure S6). By contrast, no binding was observed when PacT was incubated with the control protein FtsZ, which is a reported target of diverse bacterial toxins, indicating that PacT–Fur binding is specific (45). Next, we identified the interacting domain by using the truncated form Fur_1-83_ and found that this N-terminal DNA-binding domain could sufficiently mediate the Fur–PacT interaction. Interestingly, Fur and PacT co-expression efficiently eliminated the bacteriostatic effects of PacT (Figure 5C), indicating that Fur could in turn provide antagonistic action against PacT. Because GNAT-Xre toxins are widespread in *Pseudomonas* species, which also have highly conserved Fur proteins, we also tested the binding between GNAT toxins and Fur proteins from *P. jinjuensis* and *P. flexibilis*. Consistently, both PacT homologs could specifically bind to the DNA-binding domain of Fur (Supplementary Figure S7). Based on these results, we conclude that the specific interactions between GNAT-Xre toxins and Fur proteins seem to be a common and novel bidirectional regulatory mechanism, indicating that Fur proteins play vital roles in bacterial iron uptake.

### PacT maintains the expression levels of iron uptake genes by attenuating the promoter-binding ability of Fur

Although GNAT superfamily members can acetylate diverse substrates (i.e. from proteins to small metabolites), tRNA is the only known substrate for GNAT-type TA toxins (13). We then investigated whether PacT could acetylate Fur; however, no Fur modification was identified in the acetylation assays in vitro (Supplementary Figure S8). Thus, the specific coupling between PacT and Fur's DNA-binding domain appears to be a mechanism other than protein modification.

**Figure 6.**
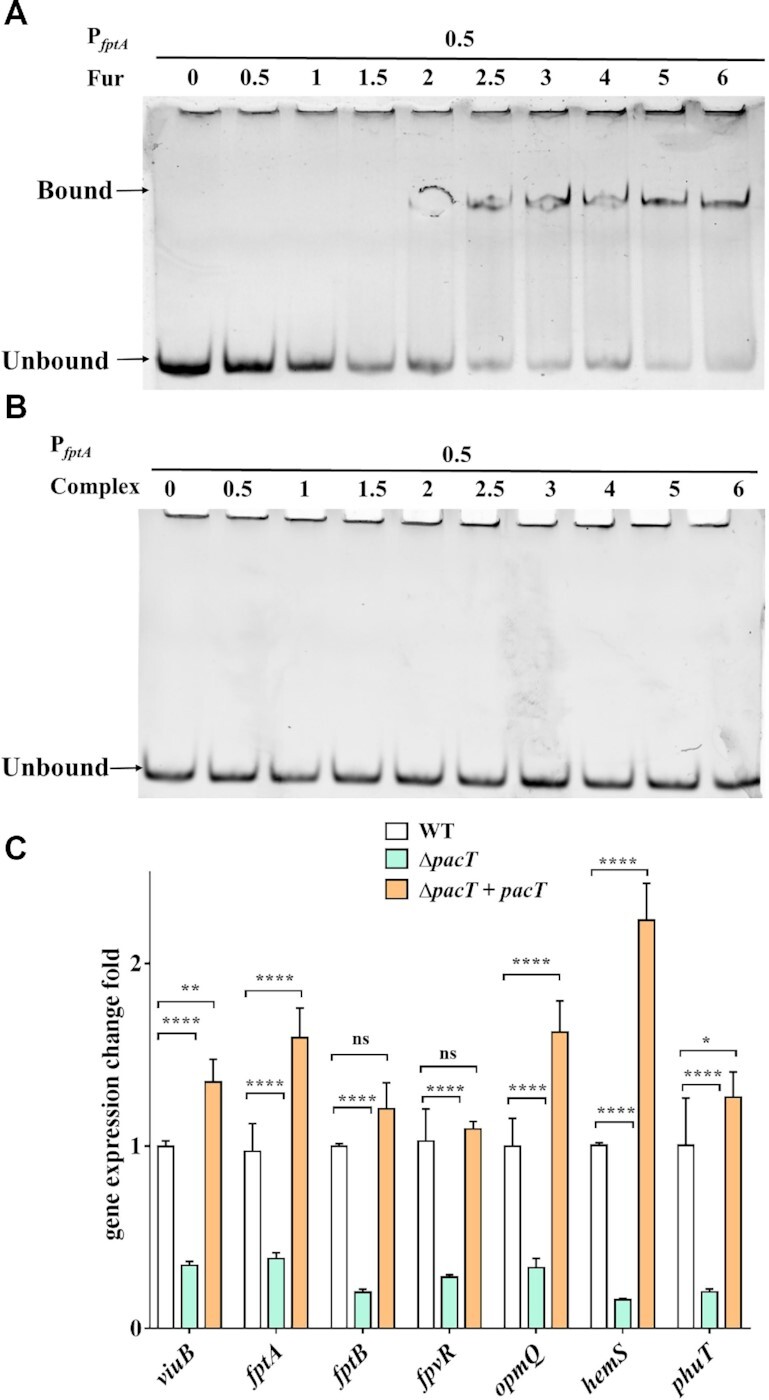
PacT attenuates DNA binding ability of Fur and maintains the normal iron uptake genes expression. (**A**) Fur could bind to the promoter region of *fptA*. The DNA was cloned from *P. aeruginosa* genome by using the primers tagged with 5’FAM and purified for EMSA assays. The final DNA concentration used in experiment was 0.5 μM, and the protein concentrations varied from 0 to 6 μM. The reaction solution was preincubated at 4°C for 30 min and electrophoresed on 10% polyacrylamide gel, then visualized under BioRad imager with Alex488 suite. (**B**) Fur-PacT complex lost DNA-binding ability, protein purification is shown in Supplementary Figure S9. The final DNA concentration used in experiment was 0.5 μM, and the protein concentrations varied from 0 to 6 μM. (**C**) The expression levels of genes involved in iron uptake decreased in Δ*pacT* strains compared to WT, this would be changed in Δ*pacT*-complemented strains. All the experiments were repeated at least three times. *P < 0.05; **P < 0.01; ***P < 0.001 by Student's *t*-test.

We reasoned that the PacT–Fur interaction could influence the DNA-binding ability of Fur. Studies have shown that Fur can recognize palindromic consensus sequences, called ‘Fur-box,’ and regulate the number of genes (46). Therefore, the promoter region of *fagA* (P*_fagA_*) that contains the Fur box was selected for electrophoretic mobility shift assays to evaluate the effects of PacT on the promoter-binding activity of Fur. Incubation of Fur with P*_fagA_* led to notably shifted DNA bands, revealing the significant protein–DNA-binding ability of Fur (Figure 6A). When complexed with PacT, Fur could no longer bind the DNA fragment, as shown by the absence of a change in DNA mobility (Figure 6B and Supplementary Figure S9). This was further confirmed by measuring the expression levels of other verified downstream targets of Fur such as *viuB*, *fptA/B, fpvR*, *opmQ, hemS* and *phuT* (15). Compared with the WT strain, the expression levels of all genes were considerably lower (decreased by 2.8- to 5.3-fold) in the Δ*pacT* strain (Figure 6C). Moreover, PacT overexpression in the Δ*pacT* strain significantly enhanced the expression levels of these genes compared with the WT strain. Based on these results, we concluded that the upregulation effects of PacT on iron-uptake-related genes are largely due to the specific interaction with Fur. Hence, PacT could inhibit the promoter-binding activity of Fur, releasing its suppression of downstream gene regulation.

### Iron starvation stimulates PacTA expression

**Figure 7.**
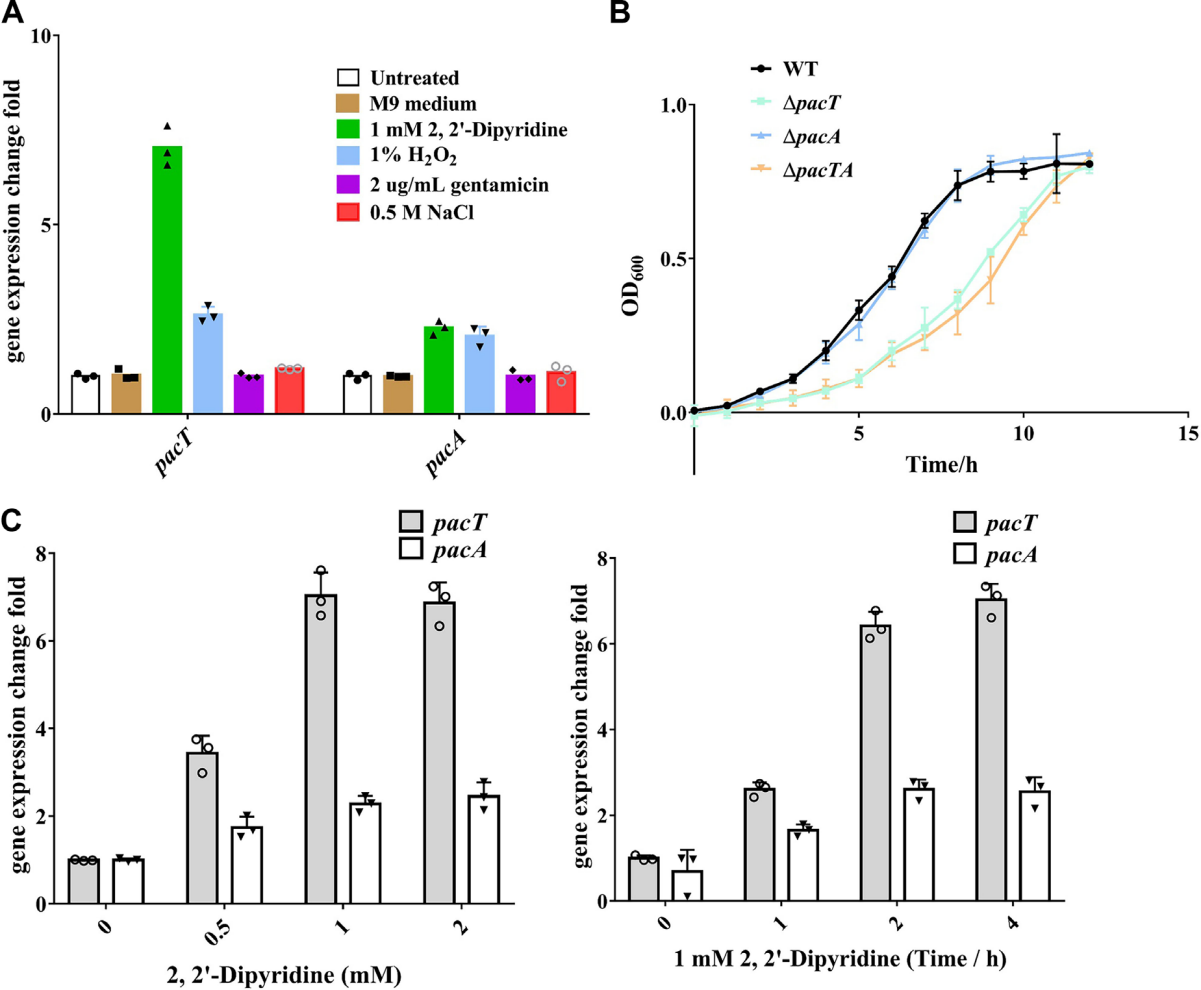
Iron starvation induces activation of the PacTA TA pair. (**A**) The expression levels of *pacT* and *pacA* were detected after the different physical and chemical stresses upon the *P. aeruginosa*. (**B**) Growth curves of WT, Δ*pacT* and Δ*pacA* strains under iron starvation. Overnight cultures of WT, Δ*pacT* and Δ*pacA* strains were reinoculated into fresh minimal succinate medium at OD_600_ = 0.05 and continued to cultivate at 37°C. (**C**) PacT functions in a time-dependent manner. qRT-PCR was performed to measure the expression levels of *pacT* and *pacA* after treatment by different concentrations of 2,2’-dipyridine. All the experiments were repeated at least three times.

Given that TA systems have been linked to stress responses (1,16), we attempted to uncover the signals inducing the expression of the PacTA TA pair. To validate the regulatory effect of the diverse stress signals on *pacTA* operon expression, *pacT* and *pacA* expression levels were detected under different physical and chemical stress conditions in *P. aeruginosa*. RT-qPCR analyses demonstrated that antibiotic treatment, osmotic condition, and carbon starvation barely altered *pacT* and *pacA* expression, whereas iron starvation or H_2_O_2_-induced oxidative stress could increase their transcription levels. *pacT* expression was significantly increased (6.2-fold) under iron-restriction conditions (Figure 7A). This suggested that the PacTA system might play a positive role under iron starvation. As expected, the Δ*pacT* strain exhibited significantly slower growth than the WT strain in the minimal succinate medium (iron-limited condition) (Figure 7B). These results reveal that *P. aeruginosa* senses iron depletion to transcriptionally control the function of the PacTA pair.

We further explored the regulatory mechanism of PacTA expression with iron-restriction stimuli. *P. aeruginosa* was treated with 0–2 mM of the ion chelator 2,2′-dipyridine for 60 min, and *pacT/pacA* expression was monitored through RT-qPCR assays (Figure 7C). *pacT/pacA* expression reached the highest levels after treatment with 1 mM 2, 2′-dipyridine. Then, whether *pacT/pacA* is induced after treatment with 1 mM 2,2′-dipyridine for 0–4 h was also determined. The results showed that extending the treatment duration to 2 h generates even higher *pacT/pacA* transcription levels (Figure 7C). Altogether, the expression effects were similarly enhanced in a time- and concentration-dependent manner, further supporting the understanding that iron starvation is a specific signal for the induction of the PacTA system.

### PacTA contributes to biofilm formation and the full virulence of *P. aeruginosa*

**Figure 8.**
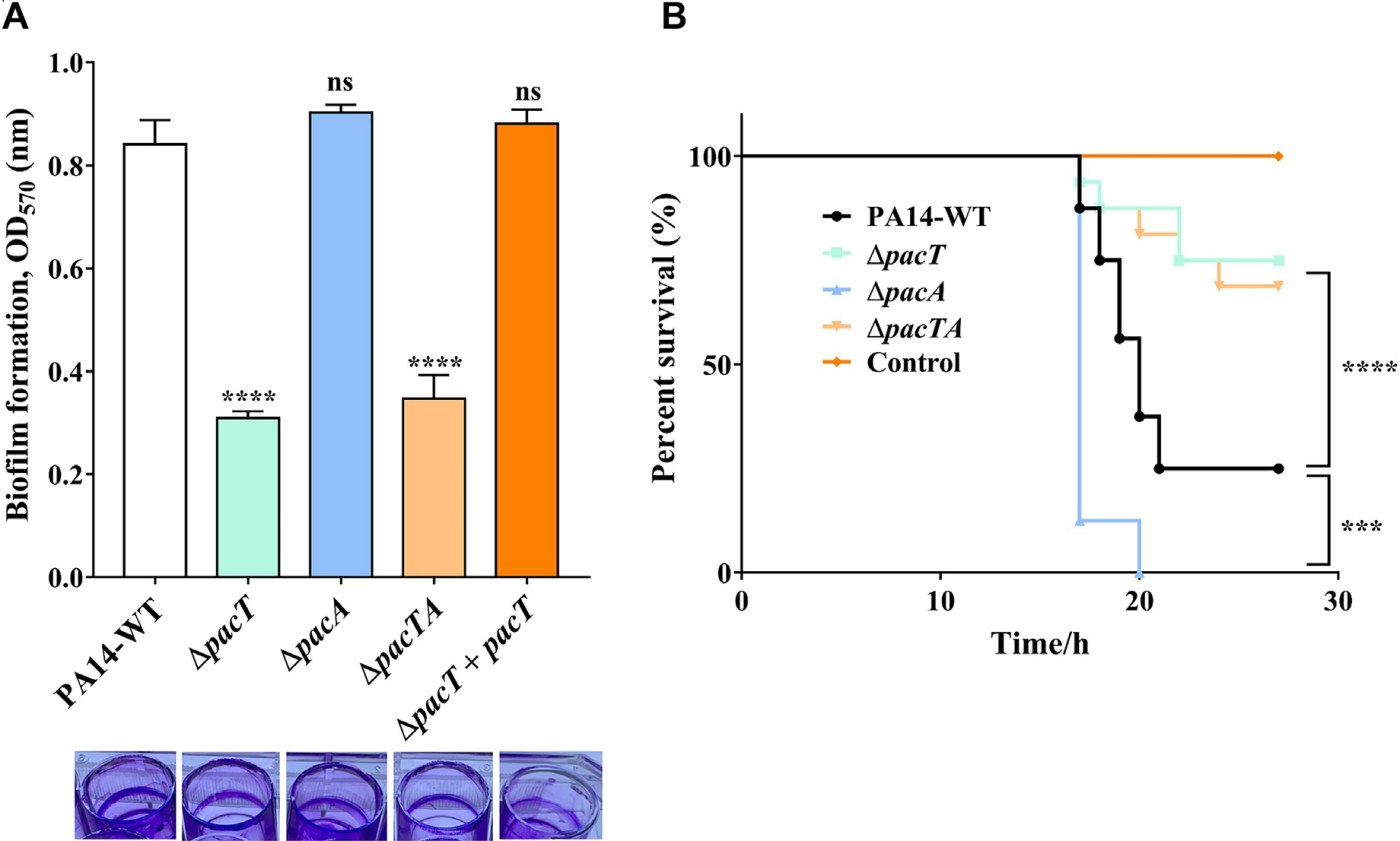
The PacTA system contributes to biofilm formation and full virulence in *P. aeruginosa*. (**A**) Biofilm production of WT, Δ*pacT* and Δ*pacA* strains. Overnight bacterial cultures were diluted at a ratio of 1:100 in LB medium and transferred into a 24-well PVC plate (Sigma) and incubated at 37°C for 48 hours. Then the biofilm was stained with crystal violet and measured at 570 nm. *P < 0.05; **P < 0.01; ***P < 0.001 and ****P < 0.0001 by One-way ANOVA statistical test. (**B**) Measurement virulence of the WT and mutant strains in a *Galleria mellonella* infection model. Each *G. mellonella* was injected with 10 μl of Pseudomonas aeruginosa dilution (1 × 10^4^ CFU/ml), and the PBS-injected larvae were the negative control. The larvae were monitored for 26 h after the infection (Mantel − Cox test for statistics, *P < 0.05).

In *P. aeruginosa*, Fur is essential for growth on solid media, and colony formation is severely impaired in *fur* mutants (47). Experimental evidence has also revealed that Fur could repress biofilm formation in many pathogens such as *Vibrio cholera*, *Yersinia pestis* and *Stenotrophomonas maltophilia* (48–50). We then investigated the impact of *pacTA* deletion on biofilm formation in *P. aeruginosa*. As expected, biofilm production by Δ*pacT and* Δ*pacTA* mutants decreased approximately 3-fold compared with that by the WT strain, whereas the Δ*pacA* exhibited no significant difference (Figure 8A).

Because iron uptake is closely related to bacterial pathogenesis and usually mediates the expression of certain virulence factors, we evaluated the contributions of the PacTA system to *P. aeruginosa* infection. The influence of Δ*pacT*, Δ*pacA* and Δ*pacTA* strains on bacterial virulence was tested in a *Galleria mellonella* infection model (28). After 16-h infection, no statistically significant difference was noted between the WT and the Δ*pacT* or Δ*pacTA* strains. However, the Δ*pacA* strain resulted in strong melanization (∼85%, Figure 8B), implying that the unrestrained basal expression of *pacT* would promote the infection process. At 24-h after infection, compared with the WT strain (70% mortality), the Δ*pacT* and Δ*pacTA* strains caused lower mortality at 25% and 30%, respectively, whereas the Δ*pacA* strain resulted in almost 100% mortality. Taken together, the PacTA TA pair plays an essential role in biofilm formation and parthenogenesis.

## DISCUSSION

**Figure 9.**
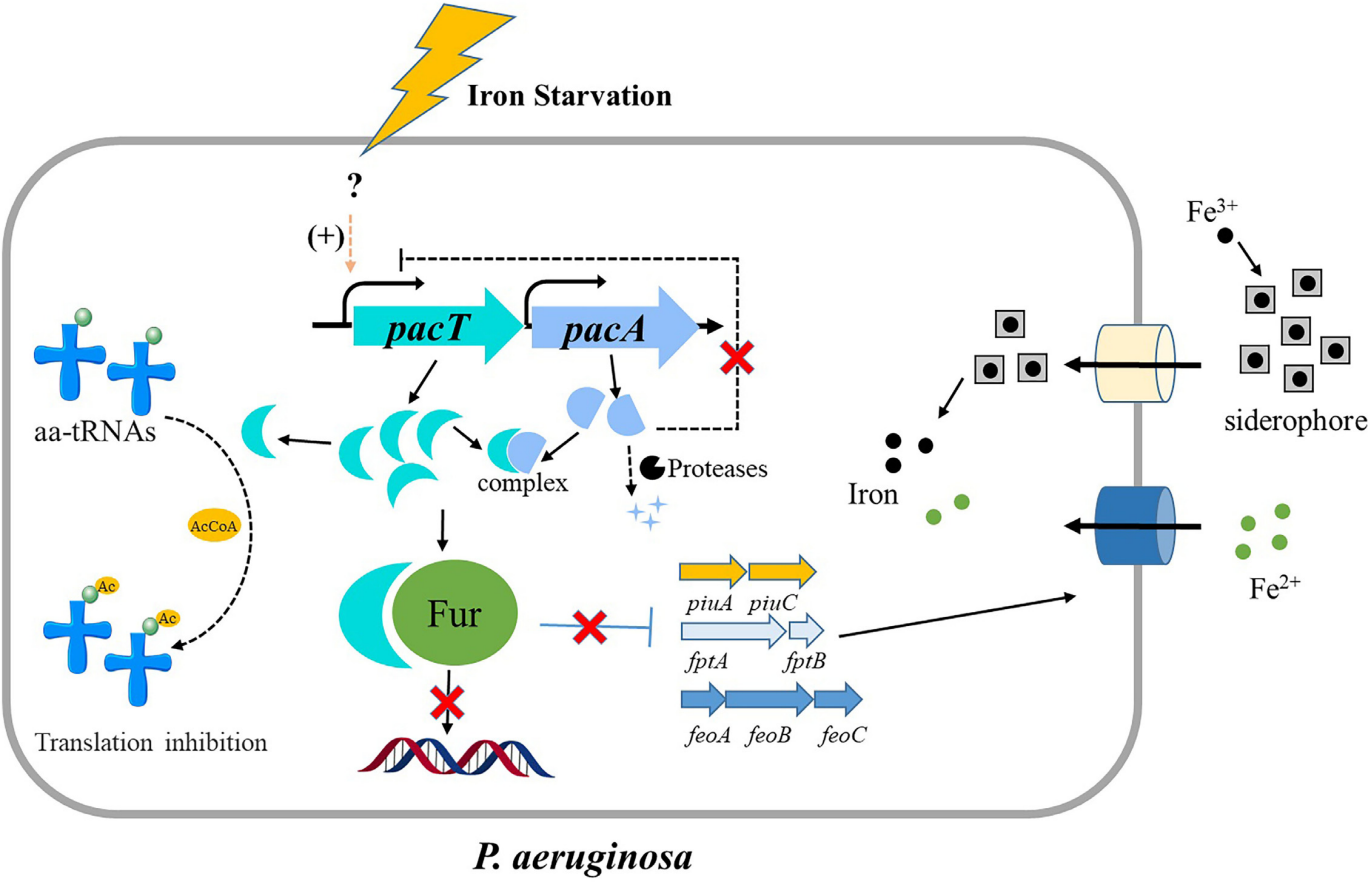
Schematic of PacTA system in control of iron uptake process in *P. aeruginosa*. During normal conditions, PacT in low expression level has limited effect on ferric uptake regulator Fur. The regulated genes such as *piuC*, *feoA* and *fptA* are repressed by Fur. Under iron-limited conditions, the expression levels of *pacT* were enhanced to attenuate the DNA-binding affinity of Fur, leading to the increasing iron acquisition genes in response to iron starvation.

In summary, we functionally characterized the putative *P. aeruginosa pa3269*/*pa3270* TA locus and verified that *pa3270* encodes a GNAT-type toxin, PacT. This toxin arrests bacterial translation and other basal metabolic processes. Previously studies on GNAT-RHH TA family also observed pronounced growth arrest after expression of the GNAT-type toxin and co-expression of its upstream antitoxin gene counteracted the toxic effect (13,51). It has been also demonstrated that the translational repression function of GNAT-type toxin depend on its acetyltransferase activity which transfers the acetyl group from acetyl coenzyme A (AcCoA) to the amine group of aa-tRNAs, subsequently resulting in the inhibition of translation (13). Considering that toxin from GNAT-Xre TA family contain a conserved CoA-binding motif and the crystal structure of PacT (PDB code: 1YRE) also verified its CoA binding ability, it is likely that acetylation reaction is involved in its repression activity, however, more studies should be carried out to characterize the biological substrate of PacT because the GNAT family has a vast variety of substrates. The main finding of our study was that we established the correlation between PacTA and iron homeostasis gene regulation (Figure 9). In normal condition, abundant cellular iron could bind to Fur as a co-repressor to improve its transcriptional repression ability. And the activity of PacT is maintained in a low state via inhibition of the cognate antitoxin PacA. Under iron starvation, the significantly increased PacT not only leads to overall translation inhibition and subsequent bacterial adaptation but also stimulates the iron uptake process by directly inhibiting the DNA-binding affinity of the negative iron acquisition regulator Fur. This accelerates the expression of iron uptake related genes and help bacteria to obtain more iron from external resources. Once enough iron has been obtained, the PacT may be inhibited at the transcriptional and protein levels by PacA, which allows the free Fur pool to incorporate the free iron to re inhibit the expression of iron-uptake genes. Therefore, PacTA provides additional repression mechanism for the strict iron regulation in *P. aeruginosa*. Previous studies mostly focused on the translation inhibition of GNAT toxins by acetylation of particular aminoacyl-tRNAs, the present study extended our understanding of the novel functional roles of type II toxins and emphasized their biological significance in the iron uptake process. To our knowledge, this is the first example of the GNAT-type toxin acting as an anti-Fur repressor. Furthermore, the relative contributions of PacTA to *P. aeruginosa* biofilm formation and virulence (Figure 8) offer a potential regulatory mechanism in which TA systems balance bacterial fitness cost during its in vivo infection.

Notably, the functional roles of toxins and antitoxins may be more sophisticated than believed (1,4,5). In *S. aureus*, the antitoxin SprF1 has a dual function. It acts as an RNA antitoxin that represses toxin expression by its 3′-end and fine-tunes overall bacterial translation by binding to ribosomes through its 5′-end (52). Interestingly, Li *et al.* reported a novel toxin–antitoxin RNA pair CreTA adjacent to CRISPR-Cas systems that functions as an addiction module preventing the loss of genes encoding the I-B CRISPR effector complex (53). In most cases, toxins from the GNAT-RHH family such as AtaT and TacT strongly inhibit protein synthesis through aa-tRNA acetylation (13,51). We here focused on the GNAT-Xre toxin of the PacTA TA system identified in *P. aeruginosa*. PacT can directly target the N-terminal HTH domain of Fur through protein–protein interactions without acetylation modification, but the binding of PacT obviously decreased the DNA-binding ability of Fur. Although it has been found that AtaT could specifically acetylate residue K206 in EspB to enhance protein stability and bacterial virulence in enterohemorrhagic *E. coli* (54), structural analysis showed that PacT (PDB: 1YRE) adapted a relatively more compact conformation, in which the substrate access channel was too narrow to accommodate protein substrate (Supplementary Figure S10). Therefore, PacT-Fur coupling is somehow analogous to the toxin-antitoxin interaction in type II TA systems such as HigBA and VapBC (31,55), suggesting that type II TA toxin may recognize various proteins other than its cognate antitoxins. This mechanism allows PacT to sense the iron starvation signal and act as an additional regulator for the iron uptake process. A similar regulatory mechanism on Fur has been reported in flagella gene repressor YdiV from *E. coli*. Under iron deficiency stress, the over-expressed YdiV and SlyD (a peptidyl-prolyl cis-trans isomerase) cooperatively act on Fur to block its DNA binding ability (50). The YdiV is necessary to activate the iron uptake gene expression in response to iron deficiency, but this is not a common mechanism in other bacteria (56). In contrast, the wide distribution of the GNAT-Xre TA family in bacterial genomes suggests that the GNAT-Xre toxin-mediated iron acquisition could be involved in various bacterial species (14)

Type II TA system has an auto-regulated modular structure: (i) toxin neutralization by antitoxin and (ii) TA operon regulation by antitoxin and TA complex via binding to the TA operon promoter region (1,31). When signals stimulate protease‐mediated degradation of antitoxin, the released toxin can execute its function. Recent reports demonstrated a significant increase in abundance of Lon protease during iron limitation in *Klebsiella pneumoniae* (57), whether the release of PacT toxin is a protease-dependent process needs further research. In addition, emerging evidences show that the expression of toxin is also activated by environmental stresses (16,31,52). In this case, the iron deficiency signal could regulate dynamic balance of PacT level in *P. aeruginosa*, while the underlying mechanism also needs to be explored in depth. To completely comprehend the mechanism and physiological activity of these TA systems, further structural analysis and characterizations of toxin targets are required.

During the past several years, numerous diverse chromosomal TA systems have been shown to participate in defense against phages, biofilm formation, persistence, and virulence in pathogenic bacteria (1,11,16,28). For example, the VapBC22 TA system in *Mycobacterium tuberculosis* modulates the expression of virulence-associated proteins and the host immune response (55). Another TA system, DarTG, in *M. tuberculosis* could catalyze reversible ADP-ribosylation of DNA for the SOS response (58). In *Xylella fastidiosa*, *mqsR* overexpression induced a series of genes to help bacterial survival under copper stress (59). Furthermore, the HigBA TA pair in *P. aeruginosa* was associated with pyocyanin production and antibiotic resistance (17). We here identified a novel regulatory mechanism of iron acquisition wherein the toxin PacT from the *P. aeruginosa* PacTA TA pair interacts with Fur to modulate gene expression and virulence. It is well known that, besides controlling metal homeostasis, Fur protein exerts a vital role in oxidative stress, biofilm formation, and virulence in bacteria (47). For instance, the *fur* mutant in *S. aureus* had a growth defect in BHI broth medium and attenuated pathogenesis in a murine infection model (60). Therefore, our finding suggested that targeting specific GNAT-Xre TA systems might be a potential therapeutic strategy for treating *P. aeruginosa* infections. Though how the iron starvation signal activates the expression or/and activation of PacT and YdiV is still unclear,

## DATA AVAILABILITY

The MS raw files and proteome sequences used in this study have been deposited to the ProteomeXchange Consortium (http://proteomecentral.proteomexchange.org) via the iProX partner repository with the dataset identifier PXD033699 (61). Atomic coordinates of the refined structures have been deposited in the Protein Data Bank (www.pdb.org) with the pdb code 8GXF, 8GXJ, 8GXK.

## Supplementary Material

gkac867_Supplemental_File
