# Supplementary material for: The novel type II toxin–antitoxin PacTA modulates Pseudomonas aeruginosa iron homeostasis by obstructing the DNA-binding activity of Fur
Source: Nucleic Acids Res. 2022 Oct 6;50(18):10586–600. doi: 10.1093/nar/gkac867 (PMC9561280; doi:10.1093/nar/gkac867)
Supplement: gkac867_Supplemental_File [file gkac867_supplemental_file.pdf]

**Supporting Information**

# The novel type II toxin–antitoxin PacTA modulates *Pseudomonas aeruginosa* iron homeostasis by obstructing the DNA-binding activity of Fur

Yingjie Song[1,3#], Siping Zhang[2#], Zirui Ye[2], Yongyan Song[3], Lin Chen[3], Aiping Tong[1], Yongxing He[2*], Rui Bao[1*]

[1] Center of Infectious Diseases, State Key Laboratory of Biotherapy, West China Hospital, Sichuan University and Collaborative Innovation Center, Chengdu 610093, China;

[2] Ministry of Education Key Laboratory of Cell Activities and Stress Adaptations, School of Life Sciences, Lanzhou University, Lanzhou 730000, China;

[3] Central Laboratory, Clinical Medical College & Affiliated Hospital of Chengdu University, Chengdu 610081, China.

[#]These authors contributed equally: Yingjie Song, Siping Zhang.

*Correspondence should be addressed to e-mail: Rui Bao: baorui@scu.edu.cn (R.B.); Yongxing He: heyx@lzu.edu.cn (Y.-X.H.)

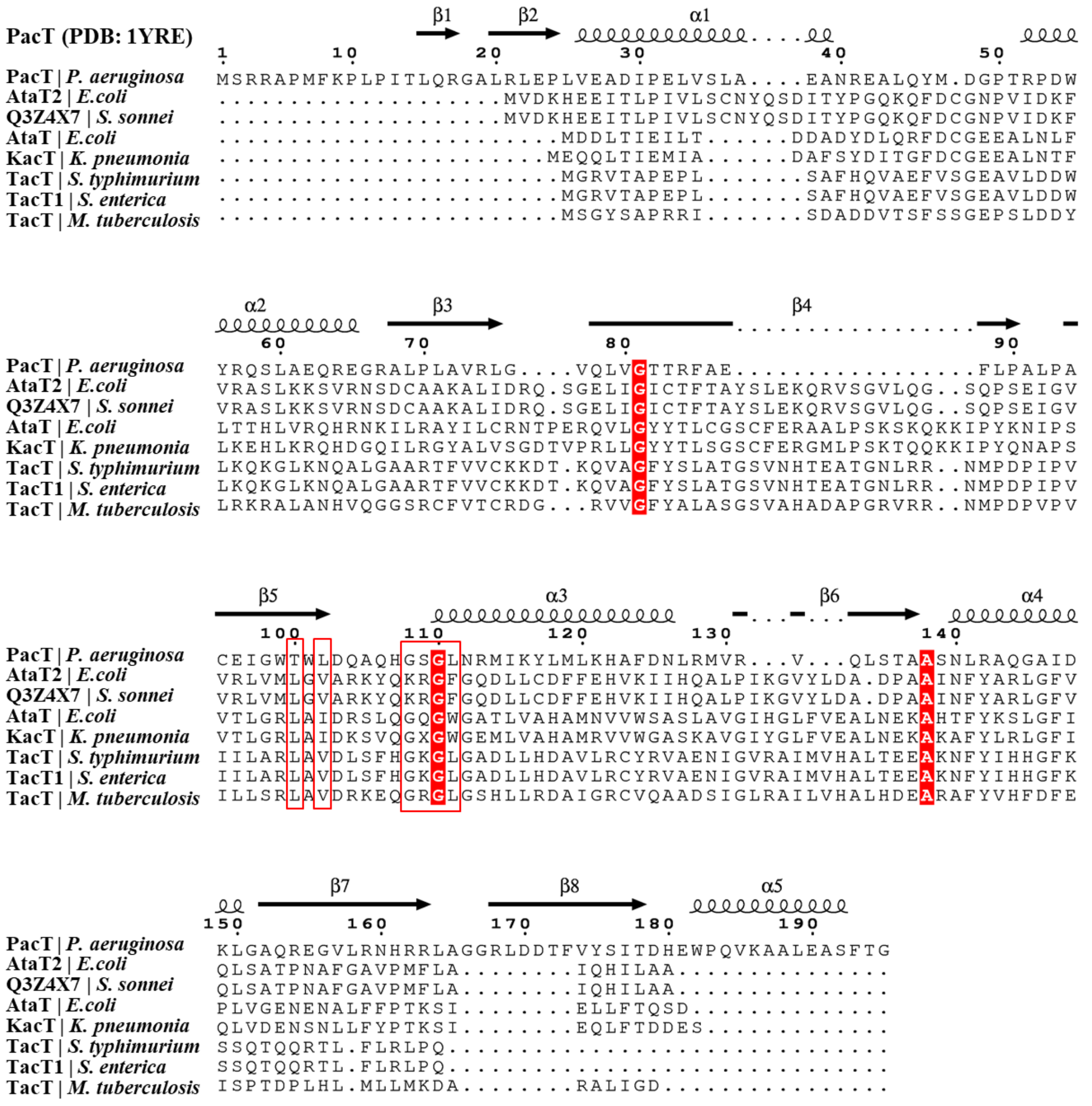


**Figure S1. Sequence alignment of PacT with other GNAT-RHH TA family toxins.** These included GNAT toxins from *E. coli* O9:H4 (strain HS), *E. coli* O157:H7, *Shigella sonnei* (strain Ss046), *K. pneumonia*, *Mycobacterium tuberculosis* (strain ATCC 25618 / H37Rv), *Salmonella typhimurium* (strain LT2 / SGSC1412 / ATCC 700720), *Salmonella enterica* subsp. enterica serovar 4, [5], 12:i:-. Amino acid sequence alignments were prepared using Clustal Omega and refined graphically using ESPript 3.0.

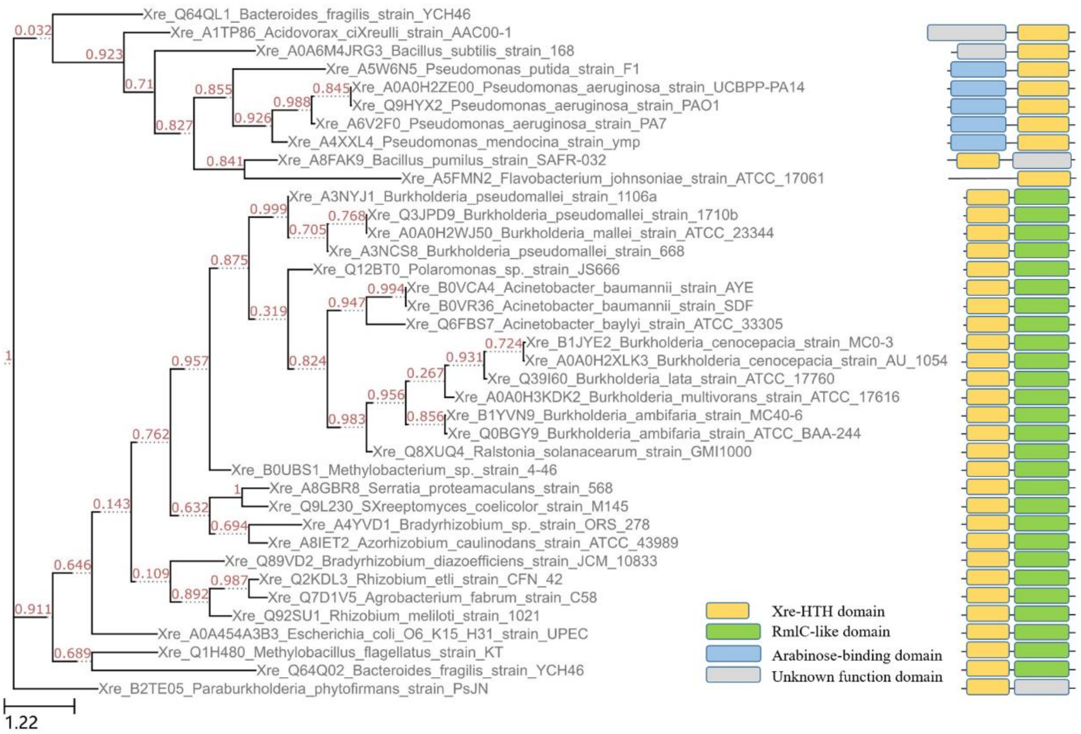


**Figure S2. Phylogenetic tree based on amino acid sequence alignment of 44 antitoxins of GNAT-Xre and GNAT-RHH TA systems obtained from TADB 2.0 database.** Amino acid sequence alignments were prepared using Clustal Omega, then the phylogenetic tree of antitoxins was generated from PhyML 3.1/3.0 aLRT and refined graphically using Phylogenetic tree viewer.

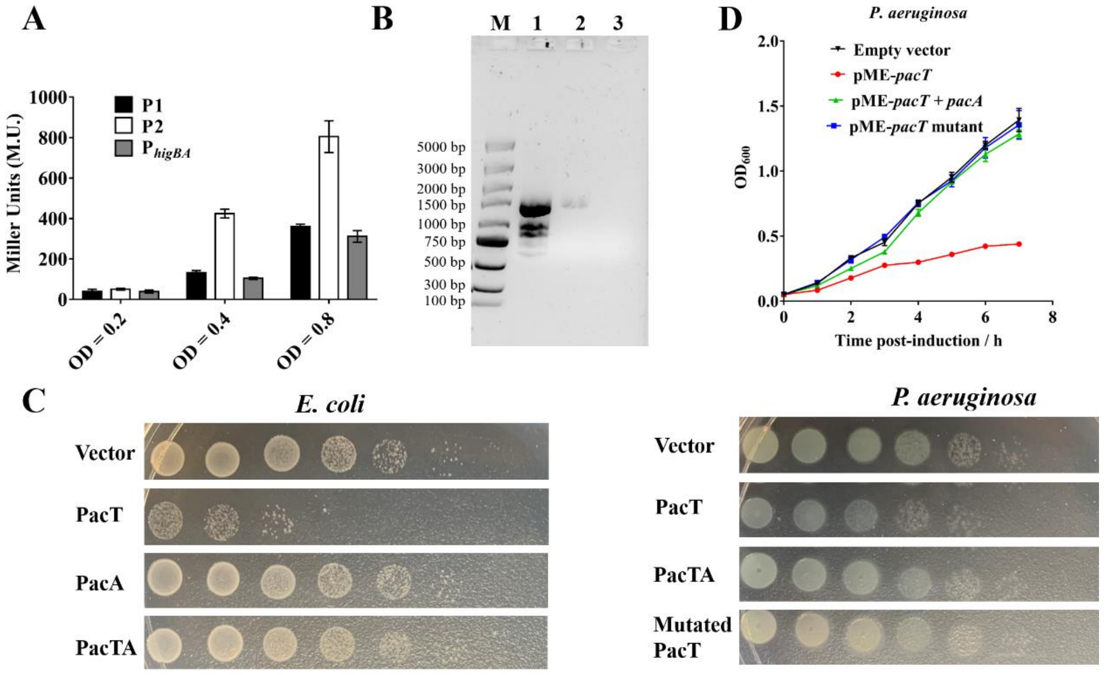


**Figure S3. Transcriptional activity of P1 and P2 in *P. aeruginosa*.** (A) The β-galactosidase reporter system was constructed to determine the promoter activities of P1 and P2, the $P_{higBA}$ served as positive control. When the *P. aeruginosa* strains carrying plasmids with P1, P2 or $P_{higBA}$ reached to $OD_{600}$ = 0.2, 0.4 and 0.8, respectively, the cells were collected and the β-galactosidase activities were measured. (B) RT-PCR was performed to determine whether *pacA* and *pacT* were co-transcribed. Line 1: total *P. aeruginosa* genome DNA, line 2: cDNA synthesized from total RNA of *P. aeruginosa*, line 3: total RNA of *P. aeruginosa*. (C) Overnight cultures of *E. coli* BL21 (DE3) carrying *pacT*, *pacA* or *pacTA* were serially diluted. The dilutions were spotted on Luria broth (LB) agar plates containing 0.1 mM IPTG. The plates were incubated at 37℃ overnight. (D) Expression of the toxin could also cause notable growth arrest of *P. aeruginosa*, while this was not observed in co-expression PacA or mutated toxin (four key resides involved in AcCoA binding, T100A, L102A, G108A, L110A). Overnight cultures of *P. aeruginosa* strains were reinoculated into fresh LB at OD600 = 0.05 and supplied with 0.3 mM IPTG, then continued to cultivate at 37℃ for growth detection. All experiments we repeated at least three times.

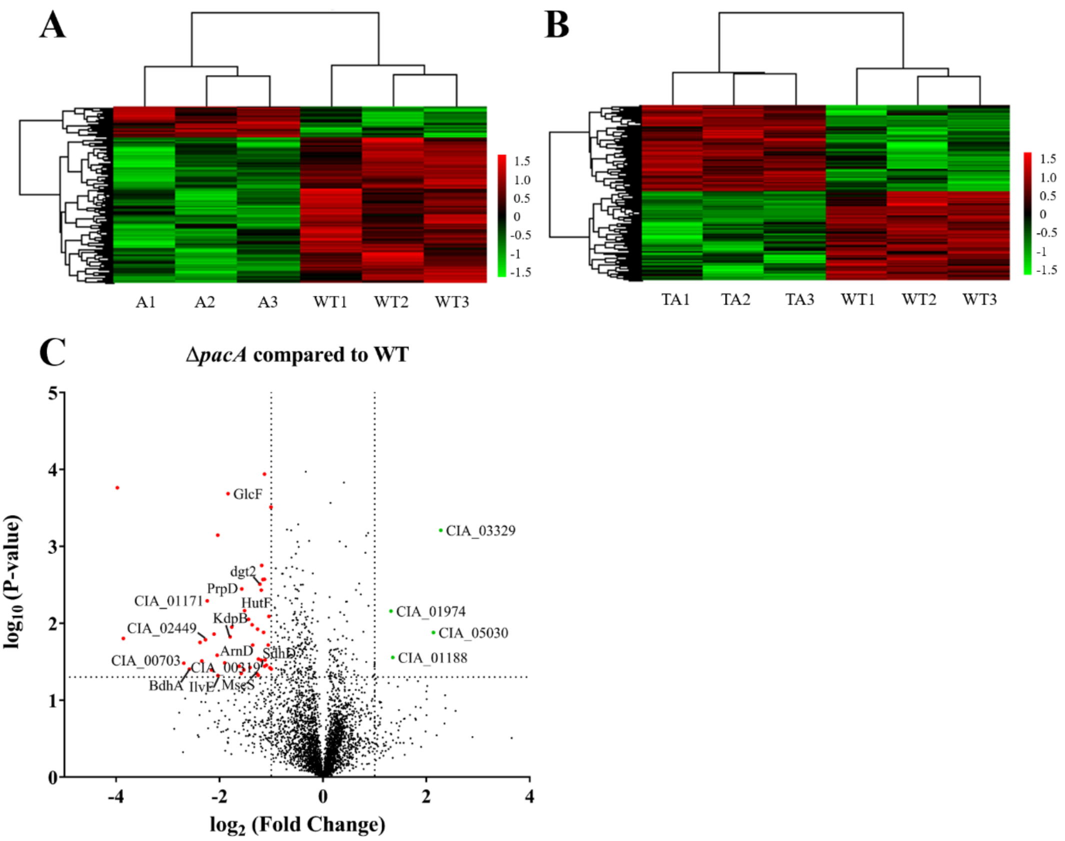


**Figure S4. Global proteomic analysis in *P. aeruginosa* Δ*pacA* and Δ*pacTA* strains.** (A) and (B) Hierarchical clustering of the z-scored extracted ion chromatogram was used to evaluate the reproducibility of the proteome quantification in Δ*pacA* and Δ*pacTA* strains. A: Δ*pacA*, TA: Δ*pacTA*. (C) Volcano plot displaying the proteomic profiles of WT and Δ*pacA* strains. The significantly up and down-regulated proteins are labeled red and green, respectively.

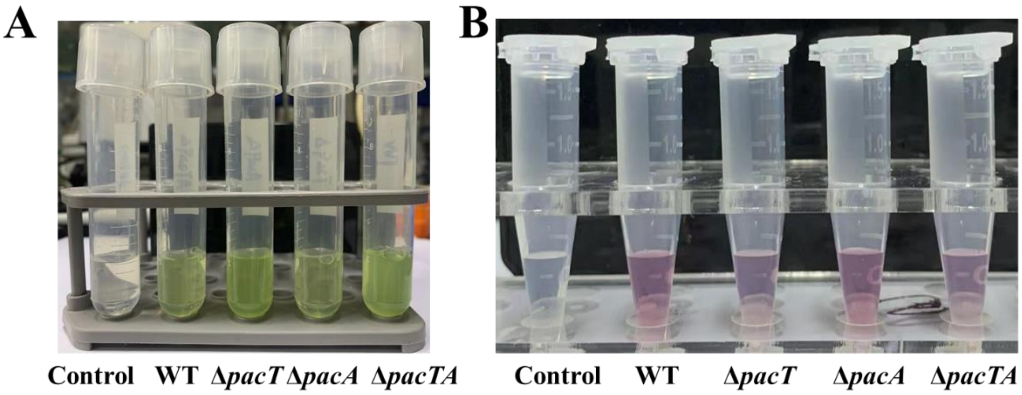


**Figure S5. Pyoverdin (A) and pyocyanin (B) production of Δ*pacT*, Δ*pacA* and Δ*pacTA* strains.** The representative color change picture corresponding to the Figure 3D. The fresh LB medium serves as negative control.

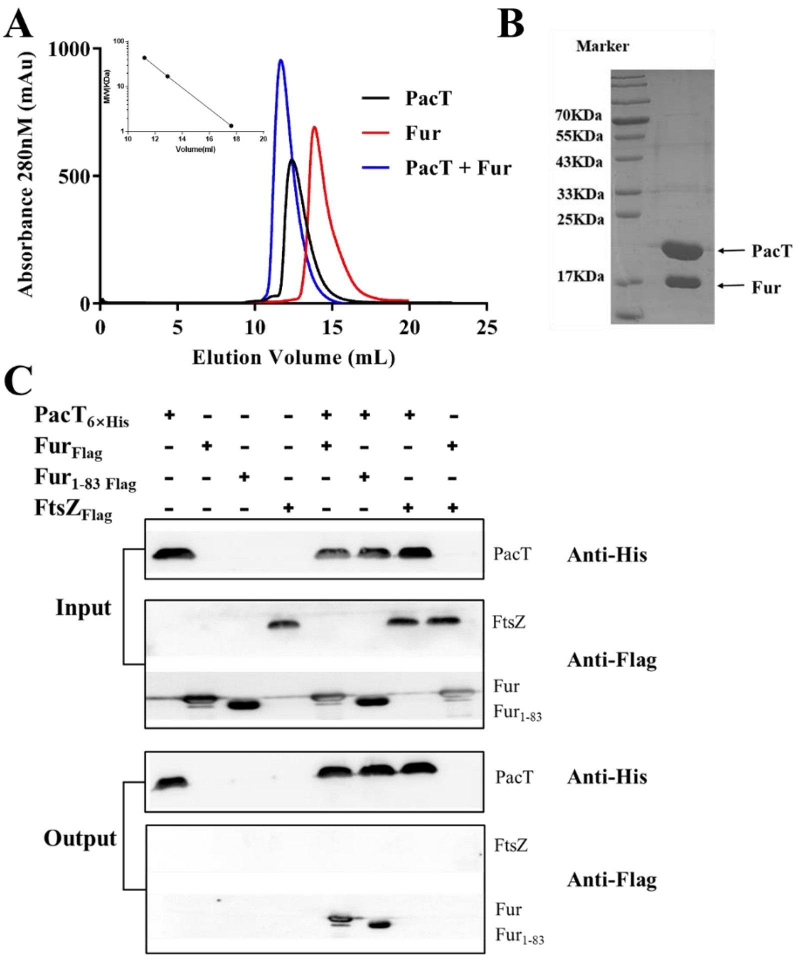


**Figure S6. The interaction between PacT and Fur.** (A) Gel filtration of PacT, Fur, and their complex in Bio-Rad SEC 70 column. The molecular weight referred to molecular weight standards (ovalbumin 44 kDa, myoglobin 17 kDa, vitamin B12 1.35 kDa, represented by three circles). (B) Purification of PacT-Fur complex. Overnight cultures of *E. coli* BL21 (DE3) carrying *pacT* and *fur* were reinoculated into fresh LB at $OD_{600}$ = 0.05 and continued to cultivate at 37°C when $OD_{600}$ reached to 0.8. Then the proteins were induced by adding 0.4 mM IPTG for 12 hours at 16°C. The cells were collected and ultrasonic crushed. The proteins were purified by $Ni^{2+}$ affinity chromatography and further analyzed by 15% SDS-PAGE. (C) Direct binding of PacT to Fur and $Fur_{1\text{-}83}$. His6-Fur was incubated with full-length Fur, $Fur_{1\text{-}83}$, or FtsZ with Flag tag, the protein complexes were captured by Ni-agarose resins and then detected by Western blotting.

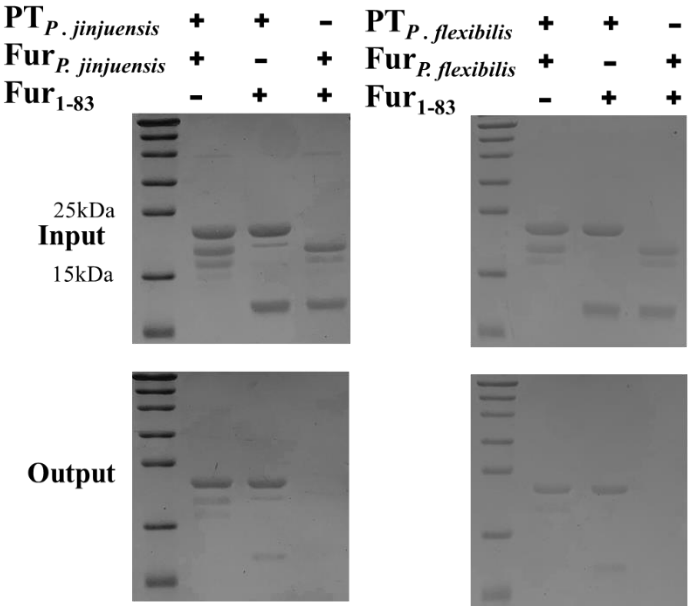


**Figure S7. The interaction between PacT homologues and fur.** Pull-down assay demonstrated that the PacT homologues from other *Pseudomonas* species also possessed the ability to bind to Fur. 20 μl His$_6$-PT$_{P.\ jinjuensis}$ or His$_6$-PT$_{P.\ flexibilis}$ (20 μM) was incubated with 10 μl full-length Fur or Fur$_{1\text{-}83}$ (35 μM) without His tag. The protein complexes were captured by Ni-agarose resins and then detected by SDS-PAGE. The FtsZ served as negative control.

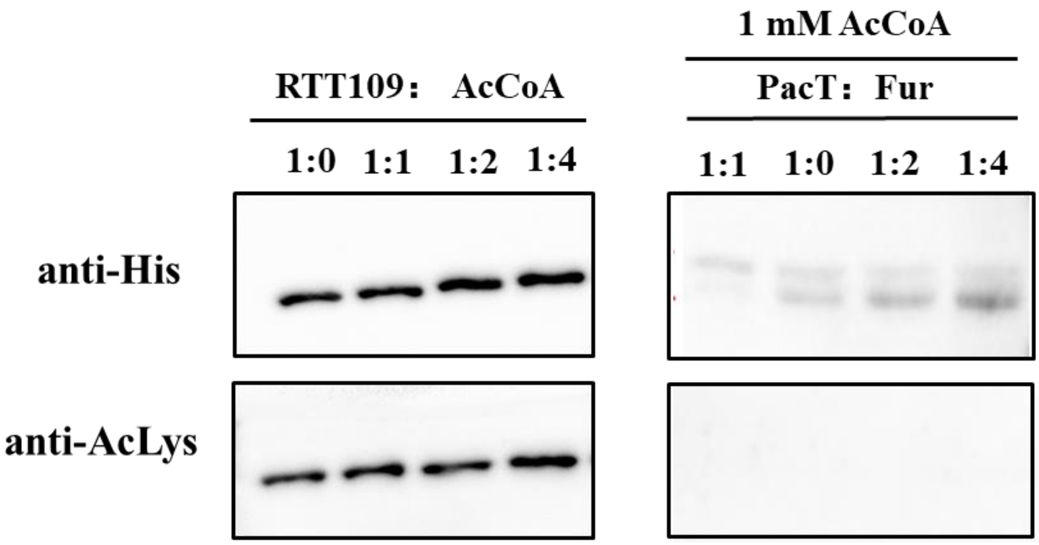


**Figure S8. WB assays demonstrated that PacT didn't acetylate Fur.** The auto-acetylation protein RTT109 from *Candida albicans* as positive control. The protein samples were firstly probed by Acetylated Lysine Antibody (Affinity, Catalogue No DF7729), then incubated with Goat Anti-Rabbit IgG (H+L) HRP (Affinity, Catalogue No S0001).

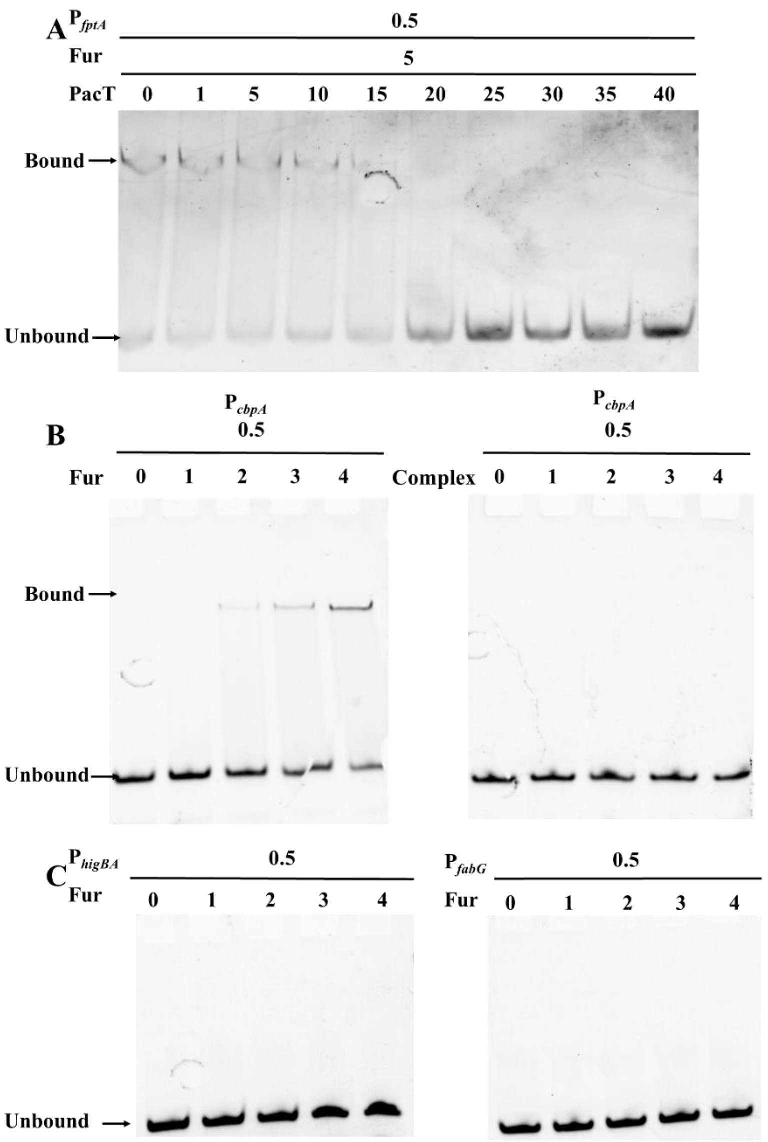


**Figure S9. PacT attenuates DNA binding ability of Fur.** (A) Gradually increasing the PacT could reduce the DNA binding ability of Fur. The final DNA concentration used in experiment was 0.5 µM, the Fur was fixed at 5 µM and the PacT concentrations varied from 0 to 40 µM. (B) Fur could also bind to the promoter regions of *cbpA* (*pa4704*), while the Fur-PacT complex lost the DNA-binding ability. The final DNA concentration used in experiment was 0.5 µM, the Fur or complex varied from 0 to 4 µM. (C) Fur showed no binding affinity to the DNA fragments of $P_{higBA}$ and $P_{fabG}$. The experiment was performed according to the method in Figure 5A and 5B. All the experiments were repeated three times.

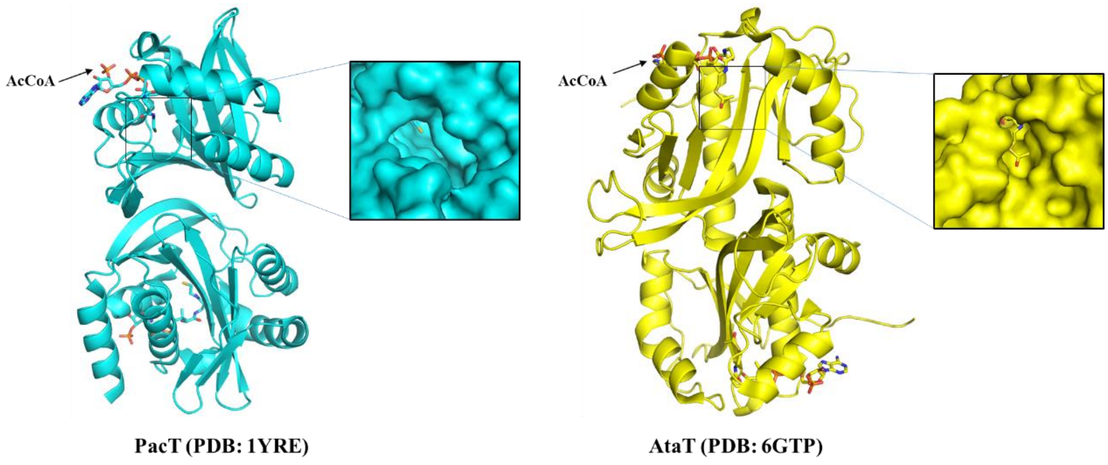


**Figure S10. Structural differences between PacT and AtaT.** Structural comparison of PacT (cyan, PDB: 1YRE) and AtaT from *E. coli* (yellow, PDB: 6GTP). Compared with AtaT, PacT adapts a relatively more compact conformation, in which the substrate access channel is too narrow to accommodate protein substrate.

**Table S1. Key resources table.**

| Reagent or Resource | Source | Identifier |
|---|---|---|
| **Bacterial cells and plasmids** | | |
| *E. coli* BL21(DE3) | Beijing Genesand Biotech Co.,Ltd | Cat# SEC19 |
| *E. coli* DH5α | Beijing Genesand Biotech Co.,Ltd | Cat# SCC01 |
| *P. aeruginosa* PA14 | Prof. Wang' Lab | N/A |
| pET-22b | This study | Song et al., (14) |
| pME6032 | This study | Song et al., (14) |
| pEX18-Gm | This study | Song et al., (14) |
| pRG970km | This study | Song et al., (14) |
| pRSFDuet-1 | This study | Song et al., (14) |
| **Chemicals** | | |
| Ni-NTA agarose | ThermoFisher | Cat# R90110 |
| ENrich SEC 70 | BioRad | Cat# 7801070 |
| **Antibodies** | | |
| anti-Flag antibody | ZEN-BIOSCIENCE | Cat# 700002 |
| anti-His tag antibody | ZEN-BIOSCIENCE | Cat# 251784 |
| Goat Anti-Rabbit IgG H&L (HRP) | ZEN-BIOSCIENCE | Cat# 511203 |
| Acetylated Lysine Antibody | Affinity | Cat# DF7729 |
| Goat Anti-Rabbit IgG（H+L）HRP | Affinity | Cat# S0001 |

**Table S2. Primers used in the work.**

| Purpose/Name | Sequence (5'-3') |
|---|---|
| **Protein Expression** | |
| **pET-22b-*pacTA*-f** | CTTTAAGAAGGAGATATACATATGATGTCGAGGAGGGCGCCCAT |
| **pET-22b-*pacTA*-r** | GTGGTGGTGGTGCTCGAGTTCGTAGATCGTCAAGGATGAGC |
| **pET-22b-*pacT*-f** | TTTAAGAAGGAGATATACATATGATGTCGAGGAGGGCGCCCATG |
| **pET-22b-*pacT*-r** | GTGGTGGTGGTGCTCGAGGCCGGTGAAGCTGGCTTCCAGCG |
| **pET-22b-*pacA*-f** | CTTTAAGAAGGAGATATACATATGGTGCCGGACGAGACGAGCGG |
| **pET-22b-*pacA*-r** | GTGGTGGTGGTGCTCGAGTTCGTAGATCGTCAAGGATGAGC |
| **pET-22b-*fur*-f** | CTTTAAGAAGGAGATATACATATGATGGTTGAAAATAGCGAAC |
| **pET-22b-*fur*-r** | GTGGTGGTGGTGCTCGAGCTTCTTCTTGCGCACGTAGAGCACC |
| **pET-22b-*ftsZ*-f** | CTTTAAGAAGGAGATATACATATGATGTTTGAACTGGTCG |
| **pET-22b-*ftsZ*-r** | GTGGTGGTGGTGCTCGAGATCGGCCTGACGACGCAGGAACG |
| **pET-22b-*ftsZ* + TEV-f** | TTTTCAGGGCCTCGAGCACCACCACCACCAC |
| **pET-22b-*ftsZ* + TEV-r** | TACAGGTTTTCCTTCTTCTTGCGCACGTAGAGC |
| **pET-22b-*fur* + TEV-f** | TTTTCAGGGCCTCGAGCACCACCACCACCAC |
| **pET-22b-*fur* + TEV-r** | TACAGGTTTTCATCGGCCTGACGACGCAGGAAC |
| **pRSFDuet-1-line1-f** | TAATGCTTAAGTCGAACAGAAAG |
| **pRSFDuet-1-line1-r** | CTGGCTGTGGTGATGATGGTGATG |
| **pRSFDuet-1-*pacT*-f** | ACCATCATCACCACAGCCAGATGTCGAGGAGGGCGCCCATG |
| **pRSFDuet-1-*pacT*-r** | CTGTTCGPPACAAGCATTATCAGCCGGTGAAGCTGGCTTCC |
| **pRSFDuet-1-line2-f** | TAATTAACCTAGGCTGCTGC |
| **pRSFDuet-1-line2-r** | ATGTATATCTCCTTCTTATPPACAAC |
| **pRSFDuet-1-*fur*-f** | AGAAGGAGATATACATATGATGGTTGAAAATAGCGAPACTCG |
| **pRSFDuet-1-*fur*-r** | GCAGCAGCCTAGGTTAATTACTPACTCTTCTTGCGCACGTAG |
| **pET-22b-*fur*1-83-f** | CTCGAGCACCACCACCACCACCACTGAGATC |
| **pET-22b-*fur*1-83-r** | ATCGGCGAGCTCGAACACGGCATG |
| **Gene knockout** | |
| **pEX18-*pacA*-upstream-f** | AACGACGGCCAGTGCCAAGCTTGCCTTGCGTCTCGAGCCGCTGG |
| **pEX18-*pacA*-upstream-r** | CTCCTTCTTAAAGTTAAACTGCCGTCTCCTCGTTCG |
| **pEX18-*pacA*-downstream-f** | GTTTAPACTTAAGAAGGAGGTCAAGCTCACATTTGC |
| **pEX18-*pacA*-downstream-r** | TTCGAGCTCGGTACCCGGGGATCACGAAGTTGATCACC |
| **pEX18-*pacT*-upstream-f** | AACGACGGCCAGTGCCAAGCTTTTGCCGTAGCGGGCGGCGATG |
| **pEX18-*pacT*-upstream-r** | CTCCTTCTTAAAGTTAAACCGACGCAAAACTCCGCTC |
| **pEX18-*pacT*-downstream-f** | GTTTAPACTTAAGAAGGAGAGCCGGTTCCGTCGCTCCTG |
| **pEX18-*pacT*-downstream-r** | TTCGAGCTCGGTACCCGGGGATACGGGCCACCGCCAACGGCTC |
| **qRT-PCR** | |
| ***pacT*-f** | TCAAGCATGCCTTCGACAAC |
| ***pacT*-r** | AGACGAAGGTGTCGTCTAGC |
| ***pacA*-f** | TGATCAACCCCGACGAAGTG |
| ***pacA*-r** | CGCCGCCATGATGGATTTC |
| ***viuB*-f** | CAACCCGCCTATCGTCTGTT |
| ***viuB*-r** | AGATCTTGATCCGCTGGTCG |

| | |
|---|---|
| ***fptA*-f** | TCAAGGACAGCCAGAACGAC |
| ***fptA*-r** | CGGTAGTCGACGCTGTAGTC |
| ***fptB*-f** | TATCTGTTGACTCGCGGGC |
| **fptB-r** | GTACCAGCAGGCTACCGC |
| ***fpvR*-f** | CGCCGAATACGCAGAAATGG |
| ***fpvR*-r** | CGGTAACATAAGCAGGGCGA |
| ***opmQ*-f** | GCCGCAGGAAGAAATCAACG |
| ***opmQ*-r** | GTAGGTACTGCAACCGGAGG |
| ***hemS*-f** | ATAATGGCGACCTGGCGAAA |
| ***hemS*-r** | TCCGGATCAAGCACGTTGAA |
| ***phuT*-f** | TACTCAAGCAGCTCGAAGGC |
| ***phuT*-r** | ACGATAGTCAAGTTCGGCGG |
| **Reporter Plasmid** | |
| **pRG970-P2-f** | GACTGACCTACCCGGGGATCCGTCGCCATAGAAGGCGATGG |
| **pRG970-P2-r** | CTCTAGAAGAAGCTTGGGATCCCGACGCAAAACTCCGCTCCC |
| **pRG970-P1-f** | GACTGACCTACCCGGGGATCCGCGCGCAGCGCGAGGGCGTG |
| **pRG970-P1-r** | CTCTAGAAGAAGCTTGGGATCCTGCCGTCTCCTCGTTCGC |
| **pRG970-seqencing-F** | ATTCAGGCTGCGCAACTG |
| **EMSA** | |
| $\mathbf{P}_{pa4471}$**-f** | CTGGTCGAGTCGATGATGG |
| $\mathbf{P}_{pa4471}$**-r** | GGCAACCTCACCAGACGATA |
| $\mathbf{P}_{pa4221}$**-f** | CCTGCGCCTCAGCGGPACTCACGC |
| $\mathbf{P}_{pa4221}$**-r** | CGTTCGAACAGCCTCGGGGGATATA |
| $\mathbf{P}_{pa4704}$**-f** | GGCCCATTCCAGAGGGCTCGCGAC |
| $\mathbf{P}_{pa4704}$**-r** | AAAGACCCGGCAAAGTGCCGGGTC |
| **RT-PCR** | |
| ***pacT*-f** | ATGTCGAGGAGGGCGCCCATG |
| ***pacA*-r** | TTCGTAGATCGTCAAGGATGAGC |

## Table S3. Data collection and refinement statistics.

| | | PacT | $PT_{P.\ jinjuensis}$ | $PT_{P.\ flexibilis}$ |
|---|---|---|---|---|
| **Space group** | | ***P*** **$4_1$ $2_1$ 2** | ***P*** **1 $2_1$ 1** | ***P*** **1 $2_1$ 1** |
| **Data collection** | *a, b, c* (Å) | 100.25, 100.25, 147.66 | 42.876, 100.687, 94.618 | 43.889, 99.013, 98.822 |
| | α, β, γ (◦) | 90, 90, 90 | 90, 96.199, 90 | 90, 101.866, 90 |
| | Wavelength | 0.9790 | 0.9790 | 0.9793 |
| | Resolution (Å) | 17.35-2.18 (2.26-2.18)[a] | 33.43-1.78 (1.85-1.78) | 42.64-3.04 (3.15-3.04) |
| | I/σI | 39.34 (1.7) | 21.8 (1.9) | 34.1 (3.2) |
| | Completeness (%) | 99.69 (99.95) | 99.76 (99.12) | 99.60 (97.29) |
| | Rmerge | 0.146 (0.441) | 0.096 (0.590) | 0.471 (0.511) |
| | Redundancy | 13.8 (6.2) | 10.1 (4.7) | 9.4 (6.5) |
| **Refinement** | Resolution (Å) | 17.35-2.18 (2.26-2.18) | 33.43-1.78 (1.84-1.78) | 42.64-3.04 (3.15-3.04) |
| | No. of reflections | 74789 (5420) | 75982 (7475) | 30920 (3289) |
| | Rwork/Rfree[b] | 0.21/0.25 | 0.21/0.25 | 0.23/0.31 |
| | **No. of atoms** | | | |
| | Protein | 747 | 747 | 748 |
| | Ligand/ion | 404 | 404 | 192 |
| | Water | 93 | 192 | 42 |
| | **B-factors(Å$^2$)** | | | |
| | Protein | 43.01 | 21.78 | 27.16 |
| | Ligand/ion | 43.00 | 31.85 | 30.10 |
| | Water | 43.80 | 25.77 | 16.96 |
| | **R.m.s. deviations** | | | |
| | Bond lengths (Å) | 0.011 | 0.014 | 0.021 |
| | Bond angles (◦) | 1.42 | 1.61 | 1.86 |
| | Ramachandran plot(favored/allowed) | 97.16/2.17 | 97.97/1.49 | 96.65/3.02 |

[a]Numbers in parentheses are statistics of the outer shell.

[b]5% of total reflections were set aside for the Rfree calculation.

**Table S4. Significant downregulated proteins in PacT-overexpression compared with control.**

| Locus in *E. coli* | Protein name and Functions | Difference | Student's T-test *p*-value |
|---|---|---|---|
| P0A8V2 | DNA-directed RNA polymerase subunit beta RpoB | -1.15771 | 0.007396 |
| P0A6Y8 | Chaperone protein DnaK | -1.25819 | 0.005223 |
| P0A7K6 | 50S ribosomal protein L19 RplS | -4.2895 | 0.036144 |
| P0AG55 | 50S ribosomal protein L6 RplF | -1.20844 | 0.015004 |
| P0A7V3 | 30S ribosomal protein S3 RpsC | -1.13428 | 0.000825 |
| P0A9D8 | 2 3 4 5-tetrahydropyridine-2 6-dicarboxylate N-succinyltransferase | -1.03575 | 0.047169 |
| P15288 | Cytosol non-specific dipeptidase | -0.87763 | 0.005906 |
| P36938 | Phosphoglucomutase | -1.9878 | 0.037696 |
| P06999 | ATP-dependent 6-phosphofructokinase isozyme 2 | -3.37628 | 0.049854 |
| P0A6F5 | 60 kDa chaperonin | -1.40557 | 0.008882 |
| P0ADW3 | Inner membrane protein YhcB | -1.08471 | 0.004314 |
| P0A7L0 | 50S ribosomal protein L1 RplA | -0.97646 | 0.00674 |
| P0ADZ4 | 30S ribosomal protein S15 RpsO | -1.06021 | 0.014747 |
| P0AC62 | Glutaredoxin 3 | -0.74499 | 0.016063 |
| P76268 | Transcriptional regulator KdgR | -3.91815 | 0.046203 |
| P21599 | Pyruvate kinase II | -0.76154 | 0.038225 |
| P0ADY1 | Peptidyl-prolyl cis-trans isomerase D | -0.92116 | 0.04286 |
| P0A6P9 | Enolase | -0.97755 | 0.03618 |
| P0AEK2 | 3-oxoacyl-[acyl-carrier-protein] reductase FabG | -1.257 | 0.049284 |
| P0A870 | Transaldolase B | -1.32053 | 0.02371 |
| P0C018 | 50S ribosomal protein L18 RplR | -1.3419 | 0.003408 |
| P08839 | Phosphoenolpyruvate-protein phosphotransferase | -1.427 | 0.00014 |
| P05852 | tRNA N6-adenosine threonylcarbamoyltransferase TsaD | -3.01309 | 0.009957 |
| P0ABB4 | ATP synthase subunit beta AtpD | -0.71214 | 0.02982 |
| P0A7R5 | 30S ribosomal protein S10 RpsJ | -1.03542 | 0.004282 |
| P67910 | ADP-L-glycero-D-manno-heptose-6-epimerase | -2.09078 | 0.035396 |
| P0A7G6 | Protein RecA | -2.82939 | 0.023987 |
| P0A953 | 3-oxoacyl-[acyl-carrier-protein] synthase 1 | -1.88532 | 0.009208 |
| P0A6P5 | GTPase Der | -1.0603 | 0.041672 |
| P0A7J3 | 50S ribosomal protein L10 RplJ | -1.24412 | 0.00345 |
| P0AC69 | Glutaredoxin 4 | -0.90583 | 0.036617 |
| P0AD61 | Pyruvate kinase I | -1.29154 | 0.019591 |
| P0A8A0 | Probable transcriptional regulatory protein YebC | -1.70191 | 0.024631 |
| P32132 | GTP-binding protein TypA/BipA | -0.92457 | 0.036075 |
| P63284 | Chaperone protein ClpB | -3.42447 | 0.029175 |
| P0AA16 | Transcriptional regulatory protein OmpR | -2.63233 | 0.037486 |
| P0A6Z3 | Chaperone protein HtpG | -1.18159 | 0.002376 |
| P68187 | Maltose/maltodextrin import ATP-binding protein MalK | -2.44104 | 0.025718 |

| | | | |
|---|---|---|---|
| P0A9P6 | ATP-dependent RNA helicase DeaD | -1.3846 | 0.021416 |
| P69797 | PTS system mannose-specific EIIAB component | -3.36572 | 0.000798 |
| P0A8L1 | Serine--tRNA ligase SerS | -1.70282 | 0.003881 |
| P00956 | Isoleucine--tRNA ligase IleS | -1.02911 | 0.024659 |
| P0A7D4 | Adenylosuccinate synthetase | -1.55992 | 0.017391 |
| P02413 | 50S ribosomal protein L15 RplO | -1.18868 | 0.032707 |
| P0A749 | UDP-N-acetylglucosamine 1-carboxyvinyltransferase | -1.22764 | 0.034652 |
| P76658 | Bifunctional protein HldE | -1.3679 | 0.012351 |
| P0A7V8 | 30S ribosomal protein S4 RpsD | -0.69797 | 0.00178 |
| P21889 | Aspartate--tRNA ligase AspS | -3.06759 | 0.040242 |
| P0A9L3 | FKBP-type 22 kDa peptidyl-prolyl cis-trans isomerase | -3.14864 | 0.012507 |
| P0A8T7 | DNA-directed RNA polymerase subunit beta RpoC | -0.81684 | 0.020461 |
| P07118 | Valine--tRNA ligase ValS | -3.02211 | 0.001343 |
| P0AGE9 | Succinate--CoA ligase [ADP-forming] subunit alpha | -3.39911 | 0.041314 |
| P33232 | L-lactate dehydrogenase | -2.28449 | 0.045089 |
| P06959 | Dihydrolipoyllysine-residue acetyltransferase component of pyruvate dehydrogenase complex | -1.1415 | 0.030352 |
| P69776 | Major outer membrane prolipoprotein Lpp | -1.79783 | 0.018343 |
| P00957 | Alanine--tRNA ligase AlaS | -0.68723 | 0.002122 |
| P0AAI5 | 3-oxoacyl-[acyl-carrier-protein] synthase 2 | -0.94902 | 0.029249 |
| P0A9Q7 | Aldehyde-alcohol dehydrogenase | -3.81438 | 0.02823 |
| P0AEX9 | Maltose-binding periplasmic protein | -2.17971 | 0.007183 |
| P00864 | Phosphoenolpyruvate carboxylase | -1.58486 | 0.044685 |
| P00350 | 6-phosphogluconate dehydrogenase decarboxylating | -1.95414 | 0.005824 |
| P77398 | Bifunctional polymyxin resistance protein ArnA | -2.53584 | 0.021762 |
| P0A910 | Outer membrane protein A | -1.05963 | 0.00495 |
| P07913 | L-threonine 3-dehydrogenase | -1.07737 | 0.021015 |
| P0CE48 | Elongation factor Tu 2 TufB | -0.92664 | 0.00177 |
| P0CE47 | Elongation factor Tu 1 TufA | -0.92664 | 0.00177 |
| P27302 | Transketolase 1 | -1.25234 | 0.005433 |
| P0A9U3 | Uncharacterized ABC transporter ATP-binding protein YbiT | -5.51883 | 0.026069 |
| P0AAG8 | Galactose/methyl galactoside import ATP-binding protein MglA | -1.12702 | 0.030994 |
| P21888 | Cysteine--tRNA ligase CysS | -5.42104 | 0.027673 |
| P33940 | Malate:quinone oxidoreductase | -2.14193 | 0.028556 |
| P37095 | Peptidase B | -2.59251 | 0.026905 |
| P15640 | Phosphoribosylamine--glycine ligase | -4.24445 | 0.026329 |
| P0ACN7 | HTH-type transcriptional repressor CytR | -2.34725 | 0.046619 |
| P0A855 | Tol-Pal system protein TolB | -5.29518 | 0.046827 |
| P42596 | Ribosomal RNA large subunit methyltransferase G RlmG | -2.30694 | 0.009712 |
| P0A6Q3 | 3-hydroxydecanoyl-[acyl-carrier-protein] dehydratase | -5.12602 | 0.040389 |
| P0AFE4 | NADH-quinone oxidoreductase subunit K | -1.41508 | 0.042344 |
| P23865 | Tail-specific protease | -3.45333 | 0.012206 |
| P17846 | Sulfite reductase [NADPH] hemoprotein beta-component | -2.5734 | 0.033802 |
| P07639 | 3-dehydroquinate synthase | -5.00337 | 0.002991 |

| | | | |
|---|---|---|---|
| P60664 | Imidazole glycerol phosphate synthase subunit HisF | -4.11963 | 0.034403 |
| P67603 | UPF0267 protein YqfB | -3.84693 | 0.002137 |
| P77529 | L-cystine transporter YdjN | -3.08936 | 0.04425 |
| P0A8W8 | UPF0304 protein YfbU | -6.09202 | 0.03833 |
| P15770 | Shikimate dehydrogenase (NADP(+)) | -1.72195 | 0.009448 |
| P0A884 | Thymidylate synthase | -2.63661 | 0.018802 |
| P0AFK0 | Metalloprotease PmbA | -7.48043 | 9.84E-05 |
| P21165 | Xaa-Pro dipeptidase | -5.80814 | 5.58E-05 |
| P0AEB7 | RutC family protein YoaB | -3.64962 | 0.034449 |
| P09551 | Lysine/arginine/ornithine-binding periplasmic protein | -3.41717 | 0.004809 |
| P0AEQ3 | Glutamine-binding periplasmic protein | -2.35786 | 0.034856 |
| P69411 | Outer membrane lipoprotein RcsF | -2.69719 | 0.015816 |
| P23871 | Ferrochelatase | -2.47503 | 0.049123 |
| P04995 | Ferrochelatase | -1.91491 | 0.023312 |
| P37340 | Ferrochelatase | -3.09606 | 0.01506 |
| P0A8D3 | Ferrochelatase | -1.05925 | 0.027319 |

**Table S5. Significant downregulated proteins in *pacA*-KO compared with WT.**

| **Locus in PA14** | **Protein name and Functions** | **Difference** | **Student's T-test *p*-value** |
|---|---|---|---|
| CIA_00136 | hypothetical protein | -3.97669 | 0.000173 |
| CIA_00319 | peptide ABC transporter substrate-binding protein | -1.19525 | 0.030119 |
| CIA_00547 | heme oxygenase | -3.86019 | 0.015679 |
| CIA_00579 | amidase | -1.04607 | 0.008113 |
| PrpD | 2-methylcitrate dehydratase | -1.57162 | 0.003566 |
| CIA_00703 | LysR family transcriptional regulator | -2.69242 | 0.033034 |
| CIA_01094 | sprT | -1.61978 | 0.036099 |
| CIA_01169 | hemolysin D | -1.00719 | 0.000309 |
| CIA_01171 | serine 3-dehydrogenase | -2.23691 | 0.005116 |
| CIA_01272 | dehydrogenase | -1.41228 | 0.037461 |
| CIA_01325 | mechanosensitive ion channel protein MscS | -1.28115 | 0.04501 |
| SdhD | succinate dehydrogenase | -1.00111 | 0.039137 |
| KdpB | potassium-transporting ATPase subunit KdpB | -1.11955 | 0.029978 |
| CIA_01656 | hypothetical protein | -1.79683 | 0.014978 |
| CIA_01805 | hypothetical protein | -1.90106 | 0.032646 |
| CIA_01880 | GNAT family acetyltransferase | -2.03374 | 0.0478 |
| BdhA | 3-hydroxybutyrate dehydrogenase | -2.58289 | 0.039356 |
| CIA_02069 | membrane protein | -1.25092 | 0.047505 |
| CIA_02113 | hypothetical protein | -1.58266 | 0.044771 |
| CIA_02205 | bifunctional glyoxylate/hydroxypyruvate reductase B | -1.12924 | 0.036125 |
| CIA_02449 | partner of the RppH RNA hydrolase | -2.37662 | 0.017682 |
| CIA_02717 | alpha/beta hydrolase | -1.13042 | 0.002676 |
| CIA_02728 | radical SAM protein | -1.5313 | 0.040208 |
| dgt2 | Nucleotide hydrolases | -1.22146 | 0.003098 |
| CIA_03490 | amino acid dehydrogenase | -2.10581 | 0.01381 |
| ArnD | 4-deoxy-4-formamido-L-arabinose-phospho-UDP deformylase | -2.04569 | 0.025952 |
| CIA_03620 | LysR family transcriptional regulator | -1.06027 | 0.019143 |
| CIA_03967 | respiratory nitrate reductase subunit delta | -1.51793 | 0.006838 |
| CIA_03968 | nitrate reductase A subunit beta | -1.76387 | 0.011192 |
| CIA_03969 | respiratory nitrate reductase subunit alpha | -1.36047 | 0.019201 |
| CIA_03970 | MFS transporter | -1.19542 | 0.003698 |
| CIA_04250 | 2,3-Butanediol catabolism dehydrogenase | -2.34275 | 0.030898 |
| CIA_04311 | phenazine biosynthesis protein PhzC | -1.13237 | 0.000115 |
| CIA_04312 | isochorismatase | -1.44084 | 0.008877 |
| CIA_04313 | anthranilate synthase | -1.37112 | 0.010473 |
| CIA_04467 | SpoVR family protein | -1.14934 | 0.013075 |
| CIA_04484 | hypothetical protein | -1.187 | 0.001767 |
| CIA_04898 | chemotaxis protein A | -1.16379 | 0.002695 |
| CIA_04903 | chemotaxis protein | -1.2665 | 0.011877 |
| CIA_04913 | AraC family transcriptional regulator | -2.27189 | 0.016311 |

| | | | |
|---|---|---|---|
| GlcF | Glycolate oxidase subunit GlcF | -1.83632 | 0.000206 |
| HutF | formiminoglutamate deiminase | -1.24723 | 0.029213 |
| IlvE | aspartate aminotransferase | -2.15599 | 0.040391 |
| CIA_05770 | major facilitator transporter | -1.09504 | 0.034896 |
| CIA_05971 | hemin degrading factor | -2.0358 | 0.000715 |
| CIA_05978 | hypothetical protein | -1.02679 | 0.03789 |

**Table S6. Significant downregulated proteins in *pacTA*-KO compared with WT.**

| Locus in PA14 | Protein name and Functions | Difference | Student's T-test *p*-value |
|---|---|---|---|
| CIA_00020 | hypothetical protein | -1.36958 | 0.026306 |
| CIA_00042 | FAD-dependent oxidoreductase | -1.56531 | 0.007561 |
| CIA_00061 | hypothetical protein | -4.90734 | 0.002948 |
| CIA_00090 | large conductance mechanosensitive channel protein MscL | -1.38507 | 0.003612 |
| CIA_00093 | hypothetical protein | -1.55557 | 0.017923 |
| CIA_00117 | glutamate dehydrogenase | -2.20083 | 0.023681 |
| CIA_00118 | cytochrome C biogenesis protein CcsA | -5.24561 | 0.001893 |
| CIA_00129 | conjugal transfer protein TraR | -2.33971 | 0.005971 |
| CIA_00136 | hypothetical protein | -4.08615 | 0.004786 |
| CIA_00151 | hypothetical protein | -2.54021 | 0.04055 |
| PilY1 | type 4 fimbrial biogenesis protein PilY1 | -1.09547 | 0.004958 |
| CIA_00158 | D-amino acid oxidase | -1.36682 | 0.006481 |
| CIA_00219 | hypothetical protein | -1.28942 | 0.026323 |
| CIA_00293 | hypothetical protein | -2.13452 | 0.011643 |
| PiuC | PKHD-type hydroxylase PiuC | -1.64708 | 0.00315 |
| CIA_00311 | peptide ABC transporter ATP-binding protein | -1.12094 | 0.009607 |
| CIA_00315 | porin | -1.43647 | 0.048417 |
| CIA_00320 | ABC transporter | -1.70801 | 0.012135 |
| CIA_00341 | carbon-nitrogen hydrolase | -1.13092 | 0.004412 |
| FumC1 | fumarate hydratase FumC1 | -1.7073 | 0.007422 |
| CIA_00353 | ribosome hibernation promoting factor HPF | -1.47688 | 0.016429 |
| CIA_00433 | hypothetical protein | -1.33355 | 0.000555 |
| CIA_00442 | nicotinate phosphoribosyltransferase | -1.26212 | 0.044028 |
| CIA_00456 | membrane protein | -1.45708 | 0.007792 |
| CIA_00457 | 3-beta hydroxysteroid dehydrogenase | -2.56248 | 0.022136 |
| FeoA | Ferrous iron transport protein FeoA | -1.03171 | 0.030984 |
| FeoB | Ferrous iron transport protein FeoB | -1.97286 | 0.035723 |
| FeoC | Ferrous iron transport protein FeoC | -1.75586 | 0.003085 |
| CIA_00467 | universal stress protein | -1.83886 | 0.000604 |
| CIA_00470 | acyl-CoA dehydrogenase | -1.66863 | 0.000702 |
| CIA_00471 | beta-lactamase | -1.53996 | 0.03378 |
| CIA_00491 | universal stress protein A | -2.24224 | 0.000167 |
| CIA_00527 | LuxR family transcriptional regulator | -2.54212 | 0.00102 |
| PigA | Heme oxygenase PigA | -3.43923 | 0.000298 |
| CIA_00579 | amidase | -2.46984 | 0.011655 |
| CIA_00630 | enoyl-CoA hydratase | -2.96693 | 0.006464 |
| CIA_00631 | enoyl-CoA hydratase | -1.82157 | 0.000156 |
| CIA_00632 | acyl-CoA dehydrogenase | -2.60618 | 0.003975 |
| CIA_00633 | methylmalonate-semialdehyde dehydrogenase | -2.57269 | 0.003223 |
| CIA_00640 | C4-dicarboxylate ABC transporter substrate-binding protein | -1.80426 | 0.003319 |

| | | | |
|---|---|---|---|
| CIA_00669 | pyrroline-5-carboxylate dehydrogenase | -1.13119 | 0.046451 |
| CIA_00682 | 3-methylitaconate isomerase | -1.62363 | 0.001742 |
| CIA_00683 | aconitate hydratase | -1.02224 | 0.002051 |
| CIA_00684 | methylcitrate synthase | -1.85753 | 0.008536 |
| CIA_00697 | activator of HSP90 ATPase | -2.66991 | 0.020947 |
| CIA_00708 | restriction endonuclease | -1.78247 | 0.000755 |
| CIA_00730 | acetate kinase | -1.71879 | 0.000737 |
| CIA_00735 | hypothetical protein | -2.50593 | 0.01455 |
| CIA_00746 | chitin-binding protein | -1.74565 | 0.001393 |
| CIA_00763 | hypothetical protein | -2.59315 | 0.022888 |
| CIA_00767 | phenylalanine 4-monooxygenase | -2.54805 | 0.0303 |
| CIA_00771 | LysR family transcriptional regulator | -2.94269 | 0.008437 |
| CIA_00793 | N-succinylglutamate 5-semialdehyde dehydrogenase | -1.36899 | 0.047926 |
| CIA_00848 | membrane protein | -1.93636 | 0.007605 |
| CIA_00859 | porin | -1.12379 | 0.010484 |
| CIA_00863 | ferritin | -1.10799 | 0.00045 |
| CIA_00900 | hypothetical protein | -1.27246 | 0.028124 |
| CIA_00902 | anthranilate synthase subunit II | -2.35352 | 0.016171 |
| CIA_00936 | glutathione S-transferase | -1.00865 | 0.017071 |
| CIA_00943 | hypothetical protein | -1.15042 | 0.001118 |
| CIA_00995 | glycosyl transferase family 1 | -1.66869 | 0.039508 |
| CIA_00997 | hypothetical protein | -1.11424 | 0.03669 |
| FliT | flagellar assembly protein FliT | -1.13914 | 0.006831 |
| CIA_01016 | hypothetical protein | -1.07644 | 0.019798 |
| CIA_01029 | hypothetical protein | -4.29508 | 0.015117 |
| CIA_01041 | molecular chaperone | -1.4764 | 0.001654 |
| CIA_01071 | dienelactone hydrolase | -2.56749 | 0.018101 |
| CIA_01079 | nitrate reductase catalytic subunit | -3.70894 | 0.02313 |
| CIA_01123 | NADPH-dependent FMN reductase | -1.28503 | 0.001643 |
| CIA_01135 | methyltransferase | -1.31352 | 0.001257 |
| CIA_01156 | hypothetical protein | -1.31843 | 0.003468 |
| AprD | peptidase AprD | -2.25015 | 6.93E-05 |
| AprE | alkaline protease secretion protein AprE | -3.89853 | 0.000397 |
| CIA_01170 | peptidase AprF | -1.45069 | 0.001298 |
| CIA_01171 | serine 3-dehydrogenase AprA | -1.79357 | 0.037017 |
| CIA_01172 | proteinase inhibitor AprL | -1.15056 | 0.013476 |
| CIA_01174 | oxidoreductase | -4.34392 | 0.000405 |
| CIA_01185 | DNA-binding protein | -2.64146 | 0.006635 |
| CIA_01237 | hypothetical protein | -2.52962 | 0.044007 |
| CIA_01253 | short-chain dehydrogenase | -2.98817 | 0.00251 |
| CIA_01260 | glutaminase | -1.91594 | 0.000885 |
| CIA_01265 | ABC transporter | -1.43858 | 0.0017 |
| CIA_01300 | hypothetical protein | -1.32741 | 0.02072 |
| CIA_01318 | ester cyclase | -2.55478 | 0.038117 |

| | | | |
|---|---|---|---|
| CIA_01323 | hypothetical protein | -1.04358 | 0.009225 |
| CIA_01334 | hypothetical protein | -1.96571 | 0.004354 |
| CIA_01338 | agmatinase | -1.67877 | 0.004528 |
| CIA_01400 | hypothetical protein | -4.84924 | 0.006035 |
| CIA_01409 | hypothetical protein | -1.83132 | 0.002033 |
| CIA_01463 | coproporphyrinogen III oxidase | -1.95971 | 0.013321 |
| CIA_01468 | (Fe-S)-binding protein | -1.22294 | 0.00274 |
| CIA_01473 | cytochrome CBB3 | -1.85846 | 0.001301 |
| CIA_01475 | peptidase S41 | -1.69232 | 0.007142 |
| CIA_01568 | NADP-dependent oxidoreductase | -1.84927 | 0.020135 |
| HsiA2 | type VI secretion protein HsiA2 | -2.23487 | 0.001854 |
| HsiB2 | hypothetical protein HsiB2 | -3.09863 | 0.000169 |
| HsiC2 | type VI secretion protein HsiC2 | -3.58678 | 0.006548 |
| ClpV1 | ClpV1 family type VI secretion ATPase | -2.94436 | 1.69E-05 |
| Fha2 | signal peptide protein Fha2 | -3.27204 | 0.002136 |
| Lip2 | type VI secretion protein Lip2 | -1.83918 | 0.008347 |
| CIA_01592 | type VI secretion protein HsiJ2 | -2.64811 | 0.008805 |
| IcmF2 | type VI secretion protein IcmF2 | -3.74053 | 0.001853 |
| Hcp2 | type VI secretion protein Hcp2 | -2.20315 | 0.005243 |
| PscL | type III secretion system protein PscL | -1.29431 | 0.039837 |
| CIA_01673 | Appr-1-p processing protein | -1.2742 | 0.023645 |
| CIA_01687 | LuxR family transcriptional regulator | -1.28993 | 0.03112 |
| CIA_01696 | hypothetical protein | -1.56177 | 0.04619 |
| CIA_01716 | universal stress protein A | -1.92046 | 0.000597 |
| CIA_01741 | enoyl-ACP reductase | -1.37994 | 0.006239 |
| CIA_01763 | short-chain dehydrogenase | -1.12128 | 0.023221 |
| CIA_01796 | 3'-kinase | -1.29889 | 0.0193 |
| CIA_01811 | peptidase | -1.32671 | 0.023433 |
| CIA_01820 | aldehyde oxidase | -1.23174 | 0.012742 |
| CIA_01831 | glutathione S-transferase | -2.05396 | 0.040382 |
| CIA_01841 | phenazine biosynthesis protein | -1.73794 | 0.001823 |
| CIA_01852 | hypothetical protein | -1.12581 | 0.039823 |
| CIA_01875 | hypothetical protein | -2.01006 | 0.005439 |
| CIA_01884 | ribose ABC transporter ATPase | -1.2487 | 0.002527 |
| CIA_01909 | membrane protein | -1.27609 | 0.01646 |
| CIA_01932 | hypothetical protein | -1.44054 | 0.036886 |
| CIA_01933 | hypothetical protein | -3.05503 | 0.04658 |
| CIA_01938 | succinyl-CoA:3-ketoacid-CoA transferase | -1.07493 | 0.01766 |
| CIA_01941 | 3-hydroxybutyrate dehydrogenase | -2.7744 | 0.047235 |
| CIA_01949 | hydroxymethylglutaryl-CoA lyase | -1.53501 | 0.000226 |
| CIA_01950 | 3-methylcrotonyl-CoA carboxylase subunit alpha | -2.05751 | 9.39E-05 |
| CIA_01951 | gamma-carboxygeranoyl-CoA hydratase | -2.20906 | 9.34E-05 |
| CIA_01952 | methylcrotonoyl-CoA carboxylase | -2.26425 | 4.02E-05 |
| CIA_01953 | isovaleryl-CoA dehydrogenase | -2.0659 | 0.001868 |

| | | | |
|---|---|---|---|
| CIA_01954 | MerR family transcriptional regulator | -1.23874 | 0.010845 |
| CIA_01963 | hypothetical protein | -4.08227 | 0.045764 |
| CIA_01986 | AraC family transcriptional regulator | -1.24048 | 0.006098 |
| CIA_01995 | LysR family transcriptional regulator | -2.78496 | 0.030716 |
| CIA_02008 | carbamoyl transferase | -1.76456 | 0.021034 |
| CIA_02042 | allophanate hydrolase | -3.26761 | 0.025252 |
| CIA_02048 | hypothetical protein | -1.93901 | 0.002377 |
| CIA_02060 | phosphoadenosine phosphosulfate sulfotransferase | -1.86541 | 0.017438 |
| CIA_02123 | (2Fe-2S)-binding protein | -2.61437 | 0.003487 |
| CIA_02124 | (2Fe-2S)-binding protein | -2.91909 | 0.003147 |
| CIA_02125 | hydrogen cyanide synthase HcnC | -2.35018 | 7.36E-06 |
| CIA_02171 | homospermidine synthase | -1.97338 | 0.013635 |
| CIA_02172 | hypothetical protein | -1.69906 | 0.023381 |
| CIA_02189 | 2-oxoisovalerate dehydrogenase subunit alpha | -2.22467 | 0.000807 |
| CIA_02190 | 2-oxoisovalerate dehydrogenase subunit beta | -2.48722 | 0.001687 |
| CIA_02191 | branched-chain alpha-keto acid dehydrogenase subunit E2 | -1.66854 | 0.000859 |
| CIA_02192 | dihydrolipoamide dehydrogenase | -2.02603 | 0.000783 |
| CIA_02201 | transcriptional regulator | -1.63413 | 9.68E-05 |
| CIA_02202 | AP endonuclease | -1.64145 | 0.001032 |
| CIA_02203 | 2-dehydro-3-deoxygluconokinase | -2.13058 | 0.011414 |
| CIA_02205 | bifunctional glyoxylate/hydroxypyruvate reductase B | -4.31049 | 0.004881 |
| CIA_02240 | chitinase | -2.82305 | 0.039835 |
| CIA_02242 | peptide synthetase | -2.33818 | 0.000518 |
| CIA_02243 | AmbD | -5.34995 | 0.001971 |
| CIA_02244 | AmbC | -3.45777 | 0.001722 |
| CIA_02245 | peptide synthetase | -1.31932 | 0.006538 |
| CIA_02271 | alkylhydroperoxidase | -1.11276 | 0.003901 |
| CIA_02318 | hypothetical protein | -6.17537 | 4.79E-05 |
| CIA_02358 | LysR family transcriptional regulator | -1.99333 | 0.031399 |
| CIA_02365 | helicase | -3.66212 | 0.02599 |
| CIA_02366 | hypothetical protein | -1.49809 | 0.000228 |
| CIA_02367 | hypothetical protein | -1.75842 | 0.001067 |
| CIA_02368 | CRISPR-associated protein Cas5 | -1.2817 | 0.009744 |
| CIA_02369 | CRISPR-associated protein cas6/csy4, subtype I-f/ypest | -1.22245 | 0.000669 |
| CIA_02381 | hypothetical protein | -4.92852 | 0.001751 |
| CIA_02391 | serine dehydratase | -1.63488 | 0.007749 |
| CIA_02392 | serine hydroxymethyltransferase | -1.25012 | 0.02694 |
| CIA_02476 | Zn-dependent protease | -1.91991 | 0.012815 |
| CIA_02499 | acyl-CoA dehydrogenase | -3.00096 | 0.000153 |
| CIA_02500 | acetyl-CoA acetyltransferase | -2.18291 | 0.001518 |
| CIA_02501 | 3-hydroxy-2-methylbutyryl-CoA dehydrogenase | -2.26485 | 0.000166 |
| CIA_02502 | AMP-binding protein | -2.81127 | 0.010271 |
| CIA_02503 | AraC family transcriptional regulator | -1.04771 | 0.011677 |
| CIA_02522 | hypothetical protein | -2.25691 | 0.011441 |

| | | | |
|---|---|---|---|
| CIA_02589 | ABC transporter substrate-binding protein | -1.4687 | 0.019667 |
| CIA_02620 | cold-shock protein | -1.60892 | 0.016355 |
| CIA_02663 | dihydropteridine reductase | -5.42912 | 2.94E-05 |
| CIA_02684 | hypothetical protein | -1.88842 | 0.000938 |
| CIA_02685 | hypothetical protein | -1.10274 | 0.02519 |
| CIA_02691 | FAD-dependent pyridine nucleotide-disulfide oxidoreductase | -1.06523 | 0.005488 |
| CIA_02708 | hypothetical protein | -2.3395 | 0.029765 |
| CIA_02717 | alpha/beta hydrolase | -4.31029 | 0.000536 |
| CIA_02807 | chemotaxis protein | -3.11351 | 0.0018 |
| CIA_02863 | thiopurine S-methyltransferase | -1.98772 | 0.025293 |
| CIA_02872 | enoyl-CoA hydratase | -1.42345 | 0.011717 |
| CIA_02877 | LysR family transcriptional regulator | -1.90502 | 0.046634 |
| CIA_02891 | hypothetical protein | -2.15367 | 0.038299 |
| CIA_02900 | terminase | -1.42072 | 0.02346 |
| CIA_02906 | hypothetical protein | -1.22539 | 0.037312 |
| CIA_02907 | ATPase AAA | -1.42298 | 0.014361 |
| CIA_02929 | GntR family transcriptional regulator | -2.05206 | 0.044008 |
| CIA_02940 | cobalt-precorrin-6A synthase | -1.12166 | 0.023037 |
| CIA_02947 | beta-lactamase | -1.26189 | 0.011838 |
| CIA_02972 | aminopeptidase | -2.38066 | 0.015153 |
| CIA_03024 | hypothetical protein | -1.94198 | 0.022074 |
| CIA_03054 | hypothetical protein | -3.12202 | 0.000602 |
| CIA_03075 | porin | -2.75708 | 0.029764 |
| CIA_03080 | deoxyguanosinetriphosphate triphosphohydrolase | -1.30799 | 0.006868 |
| CIA_03090 | peptidase | -1.225 | 0.001748 |
| CIA_03122 | ser/threonine protein phosphatase | -1.62624 | 0.028266 |
| CIA_03137 | general secretion pathway protein I | -1.74187 | 0.015671 |
| CIA_03161 | endoribonuclease L-PSP | -1.18785 | 0.004808 |
| CIA_03168 | 4-hydroxybenzoyl-CoA thioesterase | -2.89627 | 0.043871 |
| CIA_03196 | integration host factor subunit beta | -1.56217 | 0.001124 |
| CIA_03219 | transcriptional regulator | -1.78773 | 0.038533 |
| CIA_03262 | hypothetical protein | -1.26003 | 0.012549 |
| CIA_03307 | ABC transporter substrate-binding protein | -2.03433 | 0.032243 |
| CIA_03331 | N-acetyltransferase GCN5 | -8.63662 | 2.40E-05 |
| CIA_03361 | long-chain fatty acid--CoA ligase | -1.27033 | 0.013614 |
| CIA_03373 | hypothetical protein | -1.20092 | 0.012883 |
| CIA_03390 | Clp protease proteolytic subunit ClpP | -2.09239 | 3.11E-05 |
| CIA_03391 | peptide synthetase | -3.8775 | 0.000992 |
| CIA_03392 | FAD-dependent monooxygenase | -3.21041 | 8.48E-05 |
| CIA_03393 | hypothetical protein | -4.1922 | 0.000194 |
| CIA_03394 | short-chain dehydrogenase | -3.26871 | 0.0002 |
| CIA_03395 | cytochrome P450 | -3.53809 | 8.93E-06 |
| CIA_03396 | phenazine biosynthesis protein | -3.31271 | 5.96E-07 |

| | | | |
|---|---|---|---|
| CIA_03397 | 3-oxoacyl-ACP synthase | -3.86009 | 7.91E-05 |
| CIA_03399 | hypothetical protein | -4.9557 | 0.004287 |
| CIA_03401 | ADP-L-glycero-D-manno-heptose-6-epimerase | -1.86447 | 0.019245 |
| CIA_03411 | anti-anti-sigma regulatory factor | -1.18692 | 0.037656 |
| CIA_03418 | hypothetical protein | -1.46908 | 0.027527 |
| CIA_03420 | glutamate--ammonia ligase | -1.04964 | 0.008465 |
| CIA_03433 | hypothetical protein | -1.06465 | 0.04086 |
| CIA_03462 | nitrous-oxide reductase | -2.53071 | 6.88E-05 |
| CIA_03490 | amino acid dehydrogenase | -3.63555 | 0.039735 |
| CIA_03491 | hypothetical protein | -1.40981 | 0.004031 |
| CIA_03498 | enoyl-CoA hydratase | -1.99929 | 0.007559 |
| CIA_03527 | hypothetical protein | -1.46038 | 0.00981 |
| CIA_03548 | acyl-homoserine-lactone synthase | -3.25656 | 0.010183 |
| CIA_03549 | LuxR family transcriptional regulator | -2.41972 | 0.015151 |
| CIA_03550 | glycosyl transferase family 1 | -3.09823 | 0.00229 |
| CIA_03574 | putative heavy metal binding protein | -2.37905 | 0.00131 |
| CIA_03624 | 3-hydroxyisobutyrate dehydrogenase | -1.84672 | 0.002282 |
| CIA_03669 | D-xylulose 5-phosphate | -2.25701 | 0.005661 |
| CIA_03670 | beta-lactamase | -1.83661 | 0.038671 |
| CIA_03671 | cytochrome D ubiquinol oxidase subunit II | -2.59366 | 0.020498 |
| CIA_03678 | RNA polymerase sigma factor | -3.03499 | 6.45E-05 |
| CIA_03684 | S-formylglutathione hydrolase | -1.12148 | 0.024874 |
| CIA_03728 | ABC transporter ATP-binding protein | -1.66945 | 0.002351 |
| CIA_03732 | Resistance-Nodulation-Cell Division (RND) efflux transporter | -1.15607 | 0.002791 |
| CIA_03733 | RND transporter | -1.87796 | 0.008325 |
| CIA_03743 | phosphoenolpyruvate carboxylase | -1.52919 | 0.004133 |
| CIA_03744 | type 1 pili tip component | -3.83468 | 0.003376 |
| CIA_03751 | alpha/beta hydrolase | -1.08519 | 0.017311 |
| CIA_03752 | hypothetical protein | -3.63643 | 0.046096 |
| CIA_03768 | hypothetical protein | -1.28636 | 0.001311 |
| CIA_03778 | oxidoreductase | -3.09172 | 8.76E-05 |
| CIA_03779 | peptidase M4 | -2.17962 | 0.003452 |
| CIA_03875 | membrane protein | -1.50514 | 0.001791 |
| CIA_03882 | 2-isopropylmalate synthase | -1.46062 | 0.025113 |
| CIA_03885 | NADP-dependent aryl-alcohol dehydrogenase | -1.47604 | 0.028239 |
| CIA_03887 | carbon-nitrogen hydrolase | -1.34862 | 0.00502 |
| CIA_03888 | aminotransferase | -1.51403 | 0.038553 |
| CIA_03939 | isochorismatase family hydrolase | -2.65848 | 0.019247 |
| CIA_03951 | amino acid ABC transporter substrate-binding protein | -1.32493 | 0.015878 |
| CIA_03953 | AMP-binding protein | -1.14736 | 0.048977 |
| CIA_03965 | peptidyl-prolyl cis-trans isomerase | -1.04142 | 0.004608 |
| CIA_03968 | nitrate reductase A subunit beta | -2.26767 | 0.000751 |
| CIA_03969 | respiratory nitrate reductase subunit alpha | -1.56387 | 0.003779 |

| | | | |
|---|---|---|---|
| CIA_03974 | zinc-binding protein | -1.83863 | 0.006859 |
| CIA_03990 | fusaric acid resistance protein | -1.42475 | 0.036556 |
| CIA_04008 | lipid carrier protein | -2.72533 | 0.045963 |
| CIA_04009 | protease | -2.83702 | 0.047233 |
| CIA_04016 | protein tyrosine phosphatase | -2.61892 | 0.022185 |
| CIA_04019 | ATPase | -1.30597 | 0.006195 |
| CIA_04020 | copper-translocating P-type ATPase | -2.27275 | 0.000179 |
| CIA_04021 | LuxR family transcriptional regulator | -2.79737 | 0.005407 |
| CIA_04022 | hypothetical protein | -1.22371 | 0.013645 |
| CIA_04115 | MaoC family dehydratase | -1.9612 | 0.003609 |
| CIA_04122 | aldehyde dehydrogenase | -2.50175 | 0.000759 |
| CIA_04125 | ethanolamine ammonia-lyase small subunit | -2.65551 | 0.000436 |
| CIA_04166 | outer membrane protein OprG | -2.01065 | 0.025908 |
| CIA_04210 | ATPase | -1.33617 | 0.00392 |
| CIA_04230 | GntR family transcriptional regulator | -1.95486 | 0.011684 |
| CIA_04263 | LysR family transcriptional regulator | -4.39354 | 0.020493 |
| CIA_04286 | transcriptional regulator | -1.34084 | 0.005343 |
| CIA_04292 | dihydrodipicolinate synthetase | -2.03591 | 0.015568 |
| CIA_04294 | monooxygenase | -1.41378 | 0.032255 |
| CIA_04300 | D-alanine--D-alanine ligase | -1.46497 | 0.00833 |
| CIA_04308 | methyltransferase | -4.09584 | 0.00013 |
| phzA1 | phenazine biosynthesis protein PhzA1 | -3.98954 | 0.002124 |
| phzB1 | phenazine biosynthesis protein PhzB1 | -3.20833 | 4.45E-05 |
| phzC1 | phenazine biosynthesis protein PhzC1 | -3.76085 | 2.64E-05 |
| phzD1 | isochorismatase PhzD1 | -4.76232 | 0.000353 |
| phzE1 | anthranilate synthase PhzE1 | -3.94904 | 0.000303 |
| phzF1 | 2,3-dihydro-3-hydroxyanthranilate isomerase PhzF1 | -3.20583 | 0.00042 |
| phzG1 | phenazine biosynthesis protein PhzG1 | -2.74092 | 0.000229 |
| phzS | 5-methylphenazine-1-carboxylate 1-monooxygenase PhzS | -3.33539 | 4.26E-06 |
| CIA_04335 | catalase | -2.09 | 0.000219 |
| CIA_04401 | HIT family hydrolase | -2.54502 | 0.020397 |
| CIA_04451 | spermidine/putrescine ABC transporter substrate-binding protein | -3.54233 | 0.025596 |
| CIA_04464 | thiosulfate sulfurtransferase | -1.5699 | 0.040217 |
| CIA_04465 | PrkA family serine protein kinase | -2.19708 | 0.002785 |
| CIA_04484 | hypothetical protein | -5.21242 | 1.47E-06 |
| CIA_04516 | membrane protein | -1.3307 | 0.014675 |
| CIA_04532 | norD | -2.85339 | 0.014864 |
| CIA_04534 | cytochrome C | -1.90131 | 0.003696 |
| CIA_04537 | ATPase AAA | -5.13564 | 0.001659 |
| CIA_04538 | nitrite reductase | -2.37794 | 0.000322 |
| CIA_04539 | cytochrome C biogenesis protein CcsA | -2.08519 | 0.000724 |
| CIA_04540 | cytochrome C biogenesis protein DsbD | -2.36341 | 0.008479 |
| CIA_04541 | protein nirF | -2.63746 | 0.002806 |

| | | | |
|---|---|---|---|
| CIA_04542 | AsnC family transcriptional regulator | -2.89878 | 0.010422 |
| CIA_04543 | protein nirL | -3.72452 | 0.024356 |
| CIA_04545 | protein nirH | -3.4772 | 0.002217 |
| CIA_04546 | radical SAM protein | -3.93988 | 0.000621 |
| CIA_04547 | uroporphyrin-III C-methyltransferase | -3.30613 | 5.92E-05 |
| CIA_04548 | cytochrome CBB3 | -2.87167 | 0.000654 |
| CIA_04550 | acyl-CoA dehydrogenase | -1.23925 | 0.004754 |
| CIA_04559 | allophanate hydrolase | -1.09663 | 0.006247 |
| CIA_04561 | acetyl-CoA carboxylase biotin carboxylase subunit | -1.63944 | 0.012864 |
| CIA_04571 | glycine cleavage system protein R | -2.39566 | 0.008559 |
| CIA_04582 | glutathione S-transferase | -1.44407 | 0.00776 |
| CIA_04597 | ClpA/B protease ATP binding subunit | -3.04846 | 3.08E-05 |
| CIA_04610 | CoA transferase | -1.52911 | 0.000812 |
| CIA_04640 | chemotaxis protein CheB | -1.27316 | 0.041554 |
| CIA_04654 | cystathionine beta-lyase | -1.37327 | 0.024828 |
| CIA_04664 | GTPase | -3.6769 | 0.005527 |
| CIA_04690 | coniferyl aldehyde dehydrogenase | -2.24975 | 0.029048 |
| CIA_04730 | membrane protein | -2.2896 | 0.006054 |
| CIA_04751 | alpha/beta hydrolase | -1.57593 | 0.020425 |
| CIA_04753 | transcriptional regulator | -1.92318 | 0.005197 |
| CIA_04761 | aminotransferase | -1.02877 | 0.007088 |
| CIA_04766 | TetR family transcriptional regulator | -1.15414 | 0.019502 |
| CIA_04801 | hypothetical protein | -1.50011 | 0.004056 |
| CIA_04805 | hypothetical protein | -1.46489 | 0.037039 |
| CIA_04890 | nitrate ABC transporter substrate-binding protein | -1.24357 | 0.017438 |
| CIA_04897 | Fis family transcriptional regulator | -3.1001 | 0.00013 |
| CIA_04898 | chemotaxis protein A | -2.4111 | 0.004071 |
| CIA_04900 | chemotaxis protein | -2.91111 | 0.000129 |
| CIA_04903 | chemotaxis protein | -2.50368 | 0.002227 |
| CIA_04935 | polyphosphate kinase | -2.2625 | 4.91E-05 |
| CIA_04944 | omega amino acid--pyruvate aminotransferase | -1.90196 | 0.002126 |
| CIA_04985 | hypothetical protein | -1.05891 | 0.007096 |
| EvpB | EvpB family type VI secretion protein | -1.147 | 0.022991 |
| CIA_05050 | glycine/betaine ABC transporter substrate-binding protein | -1.86917 | 0.032578 |
| CIA_05053 | peptidylprolyl isomerase | -1.98277 | 0.003472 |
| CIA_05057 | quinone oxidoreductase | -1.57939 | 0.000449 |
| CIA_05153 | NrdJa | -1.88849 | 0.001126 |
| CIA_05154 | NrdJb | -2.58027 | 0.000234 |
| CIA_05169 | hypothetical protein | -1.05435 | 0.032445 |
| CIA_05174 | GNAT family acetyltransferase | -1.6746 | 0.005272 |
| CIA_05196 | GDP-mannose 4,6-dehydratase | -1.38268 | 0.029005 |
| CIA_05200 | glycosyl transferase | -1.41655 | 0.045319 |
| CIA_05210 | protease | -1.07933 | 0.001477 |
| CIA_05224 | alcohol dehydrogenase | -1.9327 | 0.0001 |

| | | | |
|---|---|---|---|
| CIA_05257 | 50S ribosomal protein L21 | -2.6145 | 0.002979 |
| CIA_05271 | hypothetical protein | -1.51284 | 0.021868 |
| CIA_05298 | hypothetical protein | -2.07387 | 0.009052 |
| CIA_05343 | omega amino acid--pyruvate aminotransferase | -1.88776 | 0.018375 |
| CIA_05354 | alanine racemase | -1.10166 | 0.022834 |
| CIA_05357 | acetyl-CoA hydrolase | -4.2308 | 0.043846 |
| CIA_05370 | hypothetical protein | -1.22977 | 0.042971 |
| CIA_05436 | proline aminopeptidase P II | -1.17032 | 0.032019 |
| CIA_05440 | hypothetical protein | -2.81684 | 0.001458 |
| CIA_05450 | type II secretion system protein E | -1.29991 | 0.01914 |
| CIA_05452 | hypothetical protein | -1.22149 | 0.000196 |
| CIA_05473 | 3-hydroxyacyl-CoA dehydrogenase | -1.46462 | 0.010006 |
| CIA_05478 | hypothetical protein | -1.08551 | 0.028596 |
| CIA_05483 | LysR family transcriptional regulator | -1.57679 | 0.01727 |
| CIA_05489 | carbamate kinase | -1.13582 | 0.002062 |
| CIA_05490 | ornithine carbamoyltransferase | -1.28811 | 0.000343 |
| CIA_05491 | arginine deiminase | -1.031 | 0.000145 |
| CIA_05544 | hypothetical protein | -1.00188 | 0.0025 |
| CIA_05551 | SAM-dependent methyltransferase | -1.28564 | 0.000984 |
| CIA_05554 | esterase | -1.93129 | 0.003171 |
| CIA_05560 | N-formimino-L-glutamate deiminase | -4.10649 | 6.26E-05 |
| CIA_05566 | urocanate hydratase | -1.21804 | 0.001383 |
| CIA_05572 | ABC transporter ATP-binding protein | -1.07648 | 0.012244 |
| CIA_05574 | imidazolonepropionase | -1.46905 | 0.031545 |
| CIA_05642 | O-acetylhomoserine aminocarboxypropyltransferase | -2.04905 | 0.007271 |
| CIA_05684 | chemotaxis protein CheY | -2.41899 | 0.045553 |
| CIA_05710 | hypothetical protein | -1.2819 | 0.009137 |
| CIA_05748 | azurin | -1.53457 | 0.001956 |
| CIA_05755 | chemotaxis protein | -1.79304 | 0.008065 |
| CIA_05771 | aldehyde dehydrogenase | -1.68903 | 0.040893 |
| CIA_05803 | urease subunit alpha | -1.64227 | 0.011875 |
| CIA_05804 | urease subunit beta | -1.64009 | 0.043064 |
| CIA_05805 | N-acetyltransferase | -1.34378 | 0.009481 |
| CIA_05806 | urease subunit gamma | -1.53132 | 0.015421 |
| CIA_05828 | diguanylate cyclase | -1.24194 | 0.022155 |
| CIA_05877 | hypothetical protein | -1.58157 | 0.034004 |
| CIA_05882 | peroxiredoxin | -1.40646 | 0.010744 |
| CIA_05887 | hypothetical protein | -2.17112 | 0.029376 |
| CIA_05894 | multidrug transporter | -6.70361 | 0.000171 |
| CIA_05976 | cAMP-binding protein | -1.87294 | 0.000361 |

**Table S7. Significant upregulated proteins in *pacTA*-KO compared with WT.**

| **Locus in PA14** | **Protein name and Functions** | **Difference** | **Student's T-test *p*-value** |
|---|---|---|---|
| CIA_00055 | cupin | 1.0416 | 0.043374 |
| LysP | lysine transporter LysP | 1.103865 | 0.013686 |
| CIA_00082 | aldehyde oxidase | 1.151569 | 0.014212 |
| CIA_00083 | (2Fe-2S)-binding protein | 1.413574 | 0.011315 |
| MexC | multidrug efflux RND membrane fusion protein MexC | 1.305199 | 0.029575 |
| CIA_00285 | DNA gyrase inhibitor | 1.491491 | 0.00049 |
| CIA_00300 | hypothetical protein | 1.664751 | 0.032008 |
| CIA_00303 | flavodoxin | 2.042562 | 0.003821 |
| MlaE | ABC transporter permease MlaE | 1.354723 | 0.035588 |
| MscS | mechanosensitive ion channel protein MscS | 1.308246 | 0.002271 |
| PitA | phosphate transporter PitA | 1.422414 | 0.024084 |
| CIA_00674 | ATP-binding protein | 2.266259 | 0.013764 |
| CIA_00725 | AraC family transcriptional regulator | 1.852829 | 0.002279 |
| CIA_00752 | hypothetical protein | 1.940698 | 0.000895 |
| CIA_00828 | transcriptional regulator | 1.751005 | 0.001606 |
| CIA_00951 | membrane protein | 1.191369 | 0.019997 |
| CIA_01013 | transcriptional regulator | 1.254119 | 0.029536 |
| CIA_01043 | zinc-binding dehydrogenase | 1.887016 | 0.00188 |
| CIA_01075 | membrane protein | 1.59619 | 0.037166 |
| DctA2 | C4-dicarboxylate ABC transporter DctA2 | 1.089303 | 0.01846 |
| CIA_01232 | LysR family transcriptional regulator | 2.279865 | 0.00772 |
| CIA_01234 | phosphonoacetaldehyde hydrolase | 1.472521 | 0.030707 |
| CIA_01240 | ubiquinol oxidase subunit II | 2.095194 | 0.028546 |
| MatE | multidrug transporter MatE | 2.112698 | 0.048523 |
| CIA_01322 | helicase | 1.384105 | 0.014321 |
| CIA_01342 | hypothetical protein | 1.229605 | 0.029637 |
| CIA_01377 | flagellar motor protein | 2.891104 | 0.034939 |
| CIA_01461 | Crp/Fnr family transcriptional regulator | 1.249964 | 0.002836 |
| CIA_01469 | cytochrome CBB3 | 1.699497 | 0.002393 |
| CIA_01572 | hypothetical protein | 2.385184 | 0.012675 |
| CIA_01612 | DNA-3-methyladenine glycosidase | 1.12807 | 0.00863 |
| CIA_01701 | CfrX protein | 2.51028 | 0.023177 |
| CIA_01728 | hypothetical protein | 1.215361 | 0.043846 |
| CIA_01958 | hemolysin D | 1.556276 | 0.006375 |
| SstT | serine/threonine transporter SstT | 1.028425 | 0.005531 |
| CIA_02107 | hemerythrin | 3.283185 | 0.042653 |
| CIA_02316 | (2Fe-2S)-binding protein | 1.486417 | 0.020297 |
| CIA_02355 | sorbosone dehydrogenase | 1.623495 | 0.017347 |
| CIA_02473 | multidrug transporter | 1.274693 | 0.011043 |
| CIA_02577 | two-component system sensor/response regulator hybrid | 1.085925 | 0.019208 |
| CIA_02718 | MerR family transcriptional regulator | 1.489918 | 0.031571 |

| | | | |
|---|---|---|---|
| CIA_03045 | hypothetical protein | 2.842059 | 0.049375 |
| CIA_03098 | transporter | 2.953616 | 0.000371 |
| CIA_03185 | glycosyl transferase | 1.667067 | 0.027904 |
| CIA_03328 | 1-acyl-sn-glycerol-3-phosphate acyltransferase | 1.971421 | 0.021795 |
| CIA_03329 | amino acid ABC transporter substrate-binding protein | 4.115509 | 0.00026 |
| CIA_03455 | DNA-binding protein | 2.490336 | 0.001319 |
| CIA_03629 | TetR family transcriptional regulator | 1.0865 | 0.01831 |
| CIA_03796 | hypothetical protein | 3.572648 | 6.07E-07 |
| CIA_03918 | permease | 1.437511 | 0.004159 |
| CIA_03931 | citrate transporter | 2.215807 | 0.001142 |
| CIA_03970 | MFS transporter | 1.38481 | 0.000885 |
| CIA_03988 | membrane protein | 1.794111 | 0.003955 |
| FecA | Fe(III) dicitrate transport protein FecA | 1.30903 | 0.006525 |
| CIA_04002 | hypothetical protein | 2.113463 | 0.038118 |
| CIA_04067 | hypothetical protein | 1.362428 | 0.013978 |
| CIA_04136 | ATPase | 1.435097 | 0.011758 |
| CIA_04146 | MFS transporter | 2.558577 | 0.032847 |
| CIA_04208 | beta-lactamase | 1.331694 | 0.000338 |
| CIA_04341 | 50S ribosomal protein L36 | 1.500072 | 0.013294 |
| CIA_04420 | hypothetical protein | 3.099889 | 0.015495 |
| CIA_04440 | putative zinc finger protein | 2.147724 | 0.016576 |
| CIA_04557 | biotin synthase | 1.203107 | 0.01409 |
| CIA_04677 | peptidoglycan transglycosylase | 1.216854 | 0.023913 |
| CIA_04696 | hypothetical protein | 1.296034 | 0.000823 |
| CIA_04775 | diguanylate cyclase | 1.144704 | 0.003368 |
| CIA_04776 | hypothetical protein | 2.018253 | 0.015815 |
| CIA_04904 | histidine kinase | 2.552666 | 0.038702 |
| CIA_04905 | hypothetical protein | 1.196599 | 0.002126 |
| CIA_04906 | hypothetical protein | 1.005548 | 0.001989 |
| CIA_04907 | diguanylate cyclase | 1.179333 | 0.026197 |
| CIA_05004 | ABC transporter ATP-binding protein | 2.977915 | 0.01529 |
| CIA_05016 | hypothetical protein | 1.846449 | 0.024246 |
| CIA_05030 | hypothetical protein | 4.087123 | 0.015892 |
| CIA_05120 | MFS transporter | 1.348864 | 0.027848 |
| CIA_05172 | membrane protein | 1.622668 | 0.017672 |
| CIA_05204 | acetyl-CoA hydrolase | 1.115473 | 0.000426 |
| CIA_05246 | hypothetical protein | 1.14233 | 0.008523 |
| CIA_05289 | phosphate ABC transporter permease | 1.141018 | 0.019219 |
| CIA_05299 | 4-hydroxybenzoate polyprenyltransferase | 1.37897 | 0.034262 |
| CIA_05310 | helicase | 1.150745 | 0.025111 |
| CIA_05365 | choline transporter | 1.408659 | 0.01443 |
| CIA_05481 | oxidoreductase | 2.414191 | 0.002009 |
| CIA_05592 | ABC transporter permease | 1.478501 | 0.019786 |
| CIA_05619 | nuclease | 2.987001 | 0.007849 |

| | | | |
|---|---|---|---|
| CIA_05742 | hypothetical protein | 1.420026 | 0.004856 |
| CIA_05782 | acyl-CoA desaturase | 1.878944 | 0.002193 |
| CIA_05783 | MFS transporter | 1.222422 | 0.030502 |
| CIA_05966 | hypothetical protein | 1.379248 | 0.008285 |
| CIA_05974 | hemin ABC transporter ATP-binding protein | 3.506082 | 0.031937 |
| CIA_05983 | glycosyltransferase | 2.849761 | 0.031711 |
